# Phytonanoparticles for food safety and preservation: potential role, evidence and challenges in antimicrobial resistance mitigation

**DOI:** 10.3389/frabi.2026.1901804

**Published:** 2026-10-26

**Authors:** Mohsin Ahmed, Bishwambhar Mishra, Sujogya Kumar Panda, Saravanan Muthupandian, Yugal Kishore Mohanta

**Affiliations:** 1 Nano-biotechnology and Translational Knowledge Laboratory, Department of Applied Biology, University of Science and Technology Meghalaya, Baridua, Meghalaya, India; 2 Department of Biotechnology, Chaitanya Bharathi Institute of Technology (CBIT), Hyderabad, Telangana, India; 3 Centre for Biotechnology, Shiksha “O” Anusandhan (Deemed to be University), Bhubaneswar, Odisha, India; 4 Department of Medical Laboratory Technology, Faculty of Applied Medical Sciences, University of Tabuk, Tabuk, Saudi Arabia; 5 Centre for Herbal Pharmacology and Environmental Sustainability, Chettinad Hospital, and Research Institute, Chettinad Academy of Research and Education, Kelambakkam, Tamil Nadu, India

**Keywords:** antimicrobial resistance, food preservation, food safety, nano-antimicrobials, phyto-nanoparticles

## Abstract

Antimicrobial resistance (AMR) is a global threat to humankind, affecting food safety, health, and the economy. Moreover, it is evolving rapidly, resulting from the misuse and overuse of antibiotics in healthcare and agriculture a result which has emerged as a One Health challenge. It alters food systems by creating threats to food safety, food security, and public health. Such resistant microorganisms have the ability to persist and spread in the food systems, animals, water, and the environment. Such microorganisms are further sustained by the production of biofilms. There is increased demand for safe and sustainable alternatives as a result of the limited applicability of conventional antibiotics and chemical preservatives. Plant-mediated phyto-nanoparticles (PNPs) are emerging antimicrobials. A PRISMA-compliant literature search and bibliometric analysis were used to identify leading research trends and gaps. This review evaluates their applicability in different aspects of food safety and preservation and in mitigating AMR. It includes major foodborne pathogens, resistance mechanisms, biofilm formation, and synthesis of bioactive nanoparticles using plants. The main antimicrobial mechanisms of these nanoparticles include the generation of reactive oxygen species (ROS), disruption of the cell membrane, release of metal ions, enzyme inhibition, DNA damage, and interference with quorum sensing. This review includes their applicability in active food packaging, edible coatings, food preservation, and food processing surfaces. This review expands existing research by including the results of real food matrices and storage studies. The review incorporates the emerging applications of artificial intelligence (AI) and machine learning (ML) in nanoparticles design, prediction of antimicrobial activities, and optimization of synthesis parameters, and trend identification. In general, PNPs have a lot of potential for sustainable approaches to food preservation. However, standardized methods, rigorous safety evaluation, AI/ML-assisted optimization, and real-food, *in vivo*, and industrial studies are needed for successful translation.

## Graphical abstract

**Figure:**
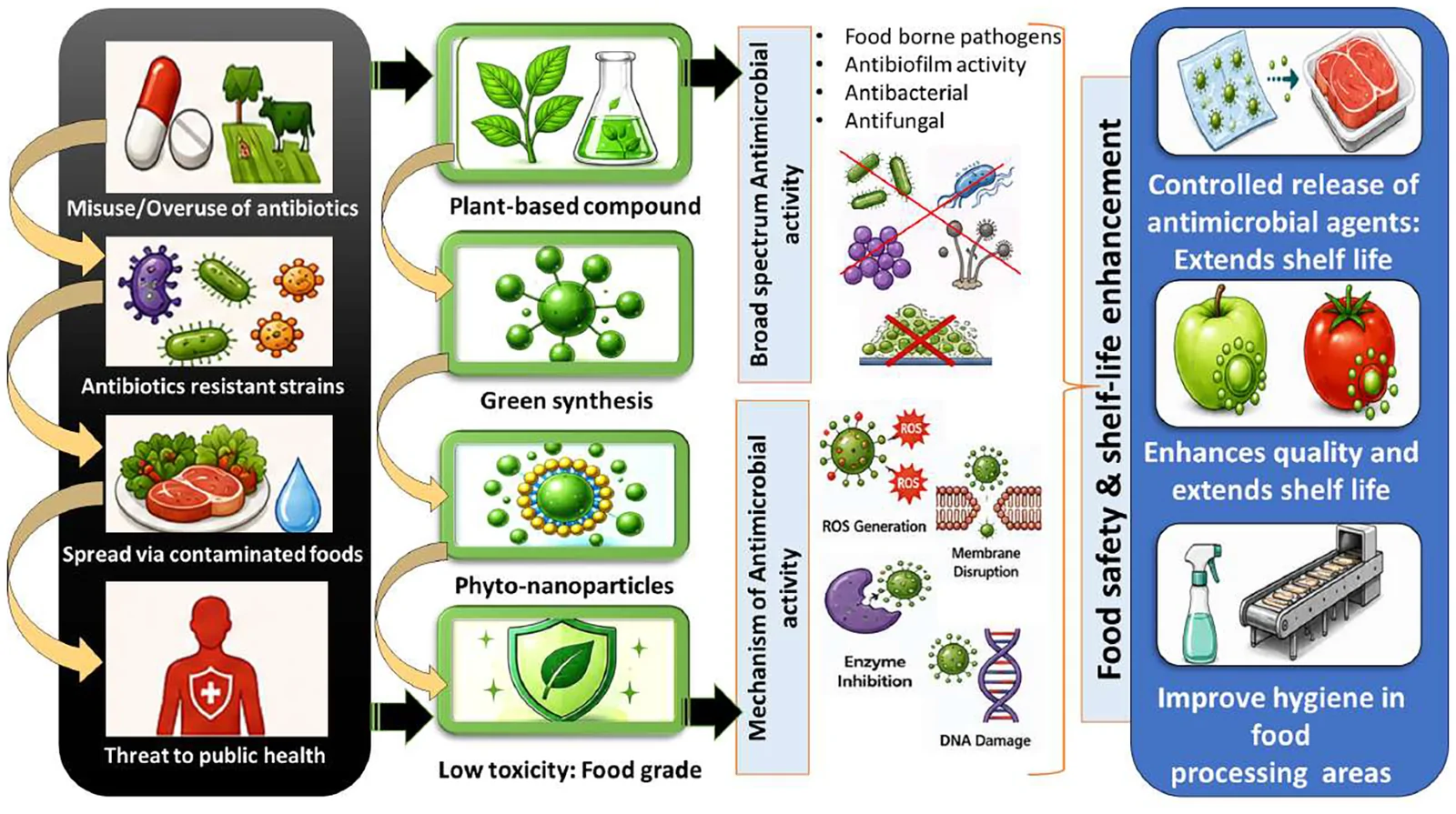

## Highlights

- Phyto-nanoparticles are promising antimicrobial materials for food safety with potential role against AMR.
- Phyto-nanoparticles exhibits broad antibacterial activity against food borne pathogens and potential antibiofilm activity.
- Nano-enabled packaging and edible coatings improve food safety and shelf life.
- Toxicity, standardization, and regulatory issues limit large-scale applications.

## 1 Introduction

In the 21st century, one of the most critical global challenges is antimicrobial resistance (AMR). Moreover, it is estimated that around 1.27 million deaths worldwide in 2019 were directly associated with AMR, while nearly 5 million deaths were directly linked to AMR, indicating a severe and growing impact on human health (Salam et al., 2023). Consequently, the excessive and exploitative use of antibiotics in human medicine, agriculture, animal husbandry, and other industries, there has been a significant rise in antibiotic resistance. As a result, several infections are very difficult to treat and control (Mann et al., 2021). This situation increases the infirmity (being diseased, unhealthy) and mortality, but also creates an economic burden worldwide on the health care system. Outside the clinical setting, in food safety and security, AMR creates a serious threat. Through contaminated water, soil, and agricultural practices, resistant pathogens can spread to people, increasing the risk of foodborne infections and outbreaks (Jangid et al., 2025).

Furthermore, the ability to control microbial contamination in the food system is reduced due to the reduced effectiveness of traditional antibiotics. The transboundary spread of resistant microorganisms further complicates mitigation efforts, underscoring the need for immediate global attention to AMR. In this context, to control AMR, there is an immediate need to develop innovative, effective, and environmentally friendly alternatives (Mubeen et al., 2021). Several innovative approaches, including green synthesis of nanoparticles and the use of plant phytochemicals as reducing agents, have shown great potential to address antimicrobial resistance. In this review, the term phytonanoparticles (PNPs) refers to metallic nanoparticles or metallic oxide nanoparticles synthesized via plant-mediated green synthesis, in which plant extracts serve as natural reducing, stabilizing, and capping agents. This review focuses mainly on plant-mediated silver (AgNPs), gold (AuNPs), Zinc oxide (ZnO NPs), and copper oxide (CuO NPs), which have been most extensively studied for the antimicrobial applications in food safety and food preservation. Nanoemulsions, polymeric nanoparticles, liposomes, nano capsules, and plant-derived extracellular vesicles are beyond the scope of this review due to their compositions, synthesis, mechanisms, and safety considerations (Barciela et al., 2023; Kaushal et al., 2024).

To solve the above-mentioned problem, global efforts focus on minimizing the misuse and overuse of antibiotics in agriculture, livestock, and food production. Several initiatives and strict regulations have been implemented by the government, such as surveillance programs and the antimicrobial stewardship initiative, which promote responsible antibiotic use worldwide to control AMR. In addition to regulatory approaches, researchers are seeking alternative antimicrobial agents to reduce reliance on traditional antibiotics (Zheng et al., 2025). However, there are several limitations of conventional food preservatives and synthetic antimicrobial agents. Overuse of chemical preservatives may lead to toxic residues, environmental pollution, alteration of food quality, and potential adverse health effects in consumers. In addition, some synthetic preservatives exhibit reduced effectiveness against foodborne microorganisms that have developed resistance, limiting their long-term use in food preservation systems. Nowadays, the growing consumer interest in natural and minimally processed foodstuffs has prompted scientists to develop new methods for their preservation; however, it is necessary to assess the safety and eco-efficiency of these methods. Natural compounds, such as plant extracts, essential oils, and bacteriocins, exhibit antimicrobial activity against the foodborne pathogen. These naturally occurring antimicrobial compounds have shown promising activity in food preservation. Still, their safety, biodegradability, and ecological effects will largely depend on their chemical structure, concentration, exposure, degradability, and toxicity (Muncke et al., 2025).

Several methods are used to control pathogenic microorganisms in food systems, with biotechnological approaches such as probiotics and the competitive exclusion method being primarily employed (van Zyl et al., 2020). Nanotechnology is another emerging technology that involves the application of enhanced antimicrobial activity and food. Several nanotechnology-based antimicrobial systems have been studied for food applications, for example, metallic nanoparticles, nanoemulsions, and nanocoatings. In this context, this review highlights only metallic/metal-oxide PNPs produced through plant-mediated processes, owing to their antimicrobial properties and the use of such nanoparticle preparation techniques to reduce dependence on conventional reducing/stabilizing agents. To prevent contamination, incorporate nanoparticles into the packaging system, thereby increasing food shelf life. However, plant-mediated synthesis reduced reliance on conventional chemicals for reduction and stabilization; nevertheless, the use of plant extracts does not provide sufficient evidence that the PNPs obtained in the process are environmentally sustainable. However, challenges related to toxicity, regulatory approval, and large-scale production still limit their widespread application. To circumvent the drawbacks mentioned above, further study and interdisciplinary approaches are required to formulate effective antimicrobial treatments against AMR, while taking into account safety, environmental, and performance (Barciela et al., 2023).

Smart nanomaterials, especially stimuli-responsive nanoparticles, are designed to enable temporally and spatially controlled, targeted delivery of antimicrobial agents (Huang et al., 2022). Broad-spectrum activity against pathogenic resistance is another emerging strategy that uses antimicrobial peptides (AMPs). There is another system known as Clustered regularly interspaced short palindromic repeats (CRISPR-Cas), a novel genetic tool that selectively targets and reduces (or eliminates) AMR bacteria. Because of its ability to specifically target and kill pathogenic bacteria, phage therapy has gained interest in recent years as an alternative to traditional antibiotics. In recent years, to accelerate the discovery of new antimicrobial compounds and the optimization of treatment strategies, artificial intelligence (AI) and machine learning have also been used. Among the emerging technologies, PNPs are being explored extensively alongside traditional antibiotics because natural compounds enhance antimicrobial effectiveness and overcome resistance mechanisms, as illustrated in **Figure 1**.

**Figure 1:**
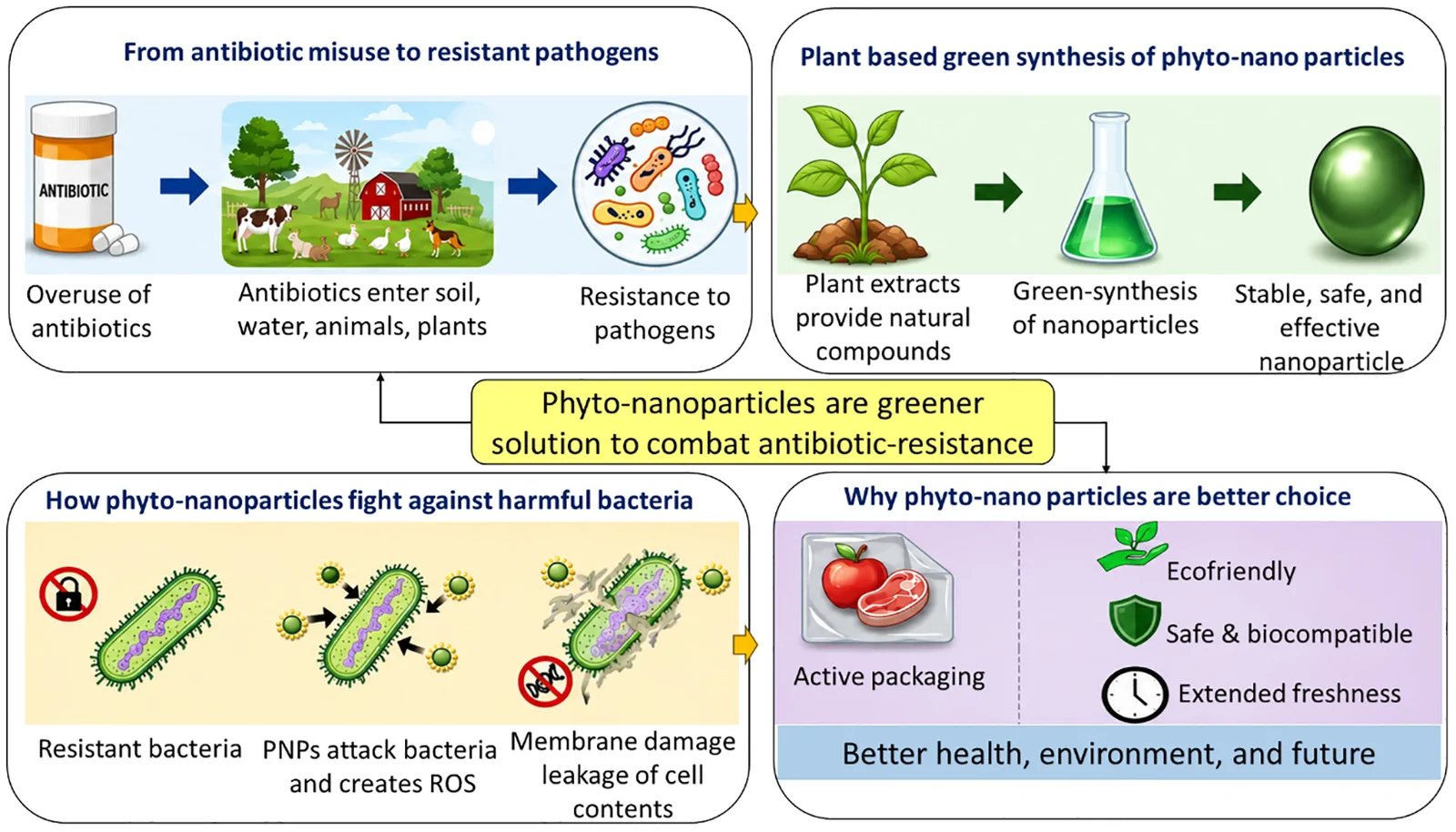
From antibiotic misuse to sustainable food protection: potential role of green-synthesized PNPs in food safety and antimicrobial resistance mitigation.

Although numerous studies have been conducted on PNPs, the current discussion emphasized deeper, more substantial, evidence-based forecasting of their extensive future utility in antimicrobial research and applications. Antimicrobial effectiveness varies across the published data; for example, there are substantial differences in plant species, phytochemical composition, synthesis, methodology, and nanoparticle physicochemical characteristics, viz. particle size, morphology, surface charge, and capping agents, etc. Consequently, it is very difficult to make direct comparisons of antimicrobial performance across the studies, and general conclusions about their effectiveness are not justified. Most available evidence is derived from laboratory-based *in vitro* experiments; however, relatively few studies have evaluated PNPs under realistic food-matrix conditions, in animal models, or in industrial food-processing environments. Under laboratory conditions, antimicrobial activity was observed, which may not directly translate to complex food systems. The practical application of PNPs for food preservation and their contribution require further standardization, validation, and comprehensive safety assessment and well-designed *in vivo* studies are needed before their large-scale implementation can be recommended (Barciela et al., 2023; Shakeel et al., 2025).

The use of a plant-mediated synthesis process may offer benefits over some traditional techniques by reducing reliance on certain synthetic reducing agents. However, the presence of such benefits does not make the PNPs environmentally sustainable; it raises issues related to resource utilization, energy use, and other aspects. In PNPs, several compounds, such as flavonoids, polyphenols, and terpenoids, further enhance antimicrobial activity by disrupting microbial cell structure and metabolic pathways (Hanna et al., 2025). In addition, the preservation system inhibits microbial growth and extends shelf life by incorporating PNPs into food packaging. Several laboratory studies have reported activity against foodborne microorganisms, which is why plant-mediated PNPs have emerged as a potential alternative antimicrobial material. Although, under laboratory conditions, PNPs exhibit antimicrobial activity against numerous foodborne microorganisms, direct evidence of their ability to reduce the evolution of resistance, eliminate resistant pathogens, or limit the dissemination of resistance genes in real food systems remains limited. So, contribution to AMR mitigation should currently be promising but not yet conclusively established (Barciela et al., 2023; Kaushal et al., 2024).

The present review evaluates the evidence on the use of PNPs as agents for food safety and preservation. Alternatively, by simply summarizing the published studies, this review studies the strength and quality of available evidence, variability in reported antimicrobial efficacy, methodological limitations, food-system applicability, safety concerns, regulatory challenges, and remaining knowledge gaps that currently limit translation into practical application. Special attention is paid to distinguishing evidence from *in vitro*, food-matrix, and *in vivo* studies to provide a balanced assessment of the realistic potential of PNPs to combat antimicrobial resistance in the food system. Recent review articles have discussed the green synthesis methods of PNPs, their properties, and antimicrobial activities. However, most of them focused on nanoparticle synthesis, biomedical applications, or food packaging individually.

On the other hand, the present review offers an integrated perspective by bridging antimicrobial resistance (AMR) in food systems and the green synthesis of PNPs, their antimicrobial mechanisms, food preservation and packaging applications, safety concerns, regulatory challenges, environmental implications, research gaps, and prospects. Collectively, these aspects provide a comprehensive and critical assessment of the opportunities, limitations, and prospects of PNPs as antimicrobial materials to enhance the food safety and mitigate antimicrobial resistance. Moreover, to highlight the novelty and scope of the present review compared with previously published articles, a comparative analysis is presented in **Table 1**.

**Table 1:** Critical comparison of recently published review articles highlighting the scope, thematic coverage, and distinctive contribution of the present review.

Primary focus	Nanomaterials covered	AMR/Resistance	Toxicity/regulatory discussion	Key scope limitation	Distinctive scope of present review	Reference
Food packaging, preservation and food safety	Polymeric, metallic nanoparticles and nanocomposites	AMR discussion limited	Brief discussion	Focuses mainly on packaging materials and nanocomposites, with less emphasis on plant-mediated nanoparticles and AMR	Integrates plant-mediated metallic and metal-oxide nanoparticles with antimicrobial mechanisms, AMR, food preservation, food packaging, safety and regulatory aspects	(Ahmad et al., 2023)
Green synthesis and antimicrobial applications	AgNPs, AuNPs, ZnO NPs, CuO NPs	AMR discussion limited	Brief discussion	Emphasis on synthesis and biomedical antimicrobial applications	Focuses on foodborne pathogens, food preservation, food packaging and AMR-related aspects	(Borehalli Mayegowda et al., 2023)
Plant-mediated nanoparticles for environmental remediation	AgNPs, AuNPs, ZnO NPs, CuO NPs, Fe_3_O_4_ NPs	AMR discussion limited	Brief discussion	Primarily addresses environmental remediation	Extends the discussion to food safety, antimicrobial mechanisms, food preservation, packaging, safety and regulation	(Dheyab et al., 2024)
Green synthesis and pharmacological applications of AgNPs	AgNPs	AMR discussion limited	Brief discussion	Focuses primarily on AgNPs and biomedical/pharmacological applications	Covers multiple plant-mediated metallic and metal-oxide PNPs with emphasis on food safety and AMR	(Dhir1 et al., 2024)

## 2 Literature search strategy and study selection

### 2.1 Prisma search strategy

A thorough review of the literature was performed on PubMed and ScienceDirect to identify studies involving plant mediated synthesis of phyto-nanoparticles (PNPs) and their role in food safety and food preservation. The synthesis of antimicrobial nanoparticles, food safety, food preservation and food packing were some of the areas researched inserting the strings [(“plant-mediated nanoparticle” OR “green-synthesized nanoparticle” OR “phyto-nanoparticle”) AND (“antimicrobial” OR antibacterial”) AND (“food safety” OR “food preservation” OR “food packaging”) AND (Antimicrobial resistance” OR “AMR”)]. Titles were screened according to predefined criteria. Studies were considered relevant if their titles contained information on plant/phyto-based nanoparticles with antimicrobial relevance and touched upon any aspect of the safety, preservation or packaging of food, including pathogens, post-harvest, or food spoilage. Relevant studies could also include the use of nanoparticles for food packaging to prolong food shelf life. The studies considered in this review, focused on the use of nanoparticles for various foods, and excluded studies on the use of nanoparticles for clinical or bio-medical purposes. Additionally, studies on the synthesis of metal or semiconductor nanoparticles, and studies focusing on the use of nanoparticles for agricultural or environmental applications were excluded. Background and review articles were also excluded.

The search returned 342 articles; of which 196 and 146 were found on PubMed and ScienceDirect, respectively. A total of 2 duplicate records were found and removed after the search. The remaining 340 records underwent a preliminary assessment based on title. Records which did not involve the synthesis of antimicrobial nanoparticles using plants, food safety and preservation, packaging as well records which were background or review records and studies involving food borne pathogens were excluded. A total of 47 and 7 records from PubMed and ScienceDirect respectively were included in the next phase of the assessment where the first 3 records from ScienceDirect were further assessed for eligibility. In **Figure 2**, the complete literature selection process is illustrated in accordance with to the PRISMA 2020 (**Supplementary Files 1**, **2**).

**Figure 2:**
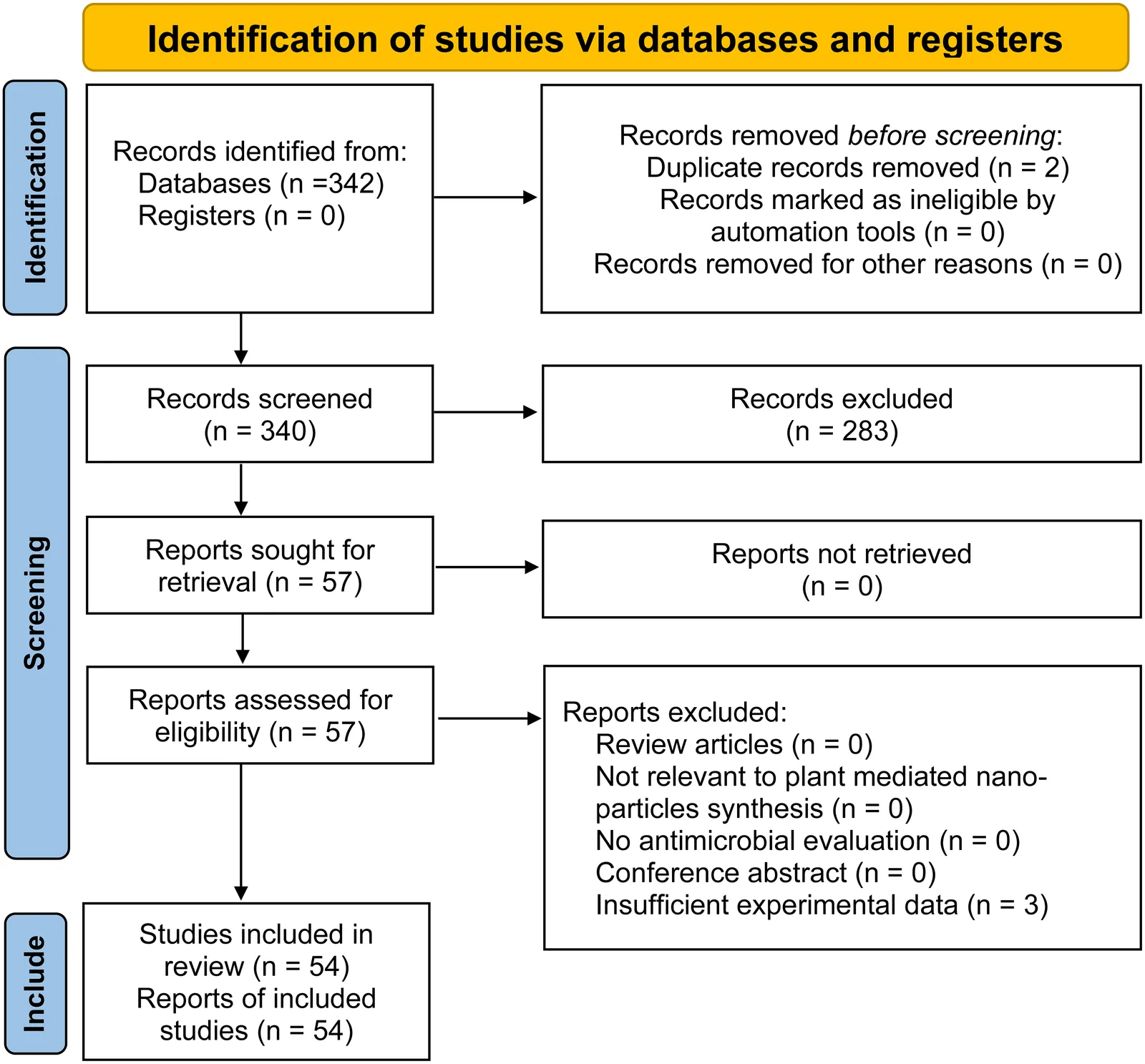
PRISMA 2020 flow diagram illustrating the literature search, screening, eligibility, assessment, and final selection of studies included in this review.

### 2.2 Bibliometric analysis of research trends on PNPs using VOSviewer

A bibliographic analysis was conducted using VOSviewer to examine global research trends in PNPs and antimicrobial applications. All this analysis was performed using VOSviewer which allows visualization of a keyword co-occurrence network and the identification of research gaps. The keyword co-occurrence network showed four main groups, each of them describing a distinct thematic area (**Figure 3**).

**Figure 3:**
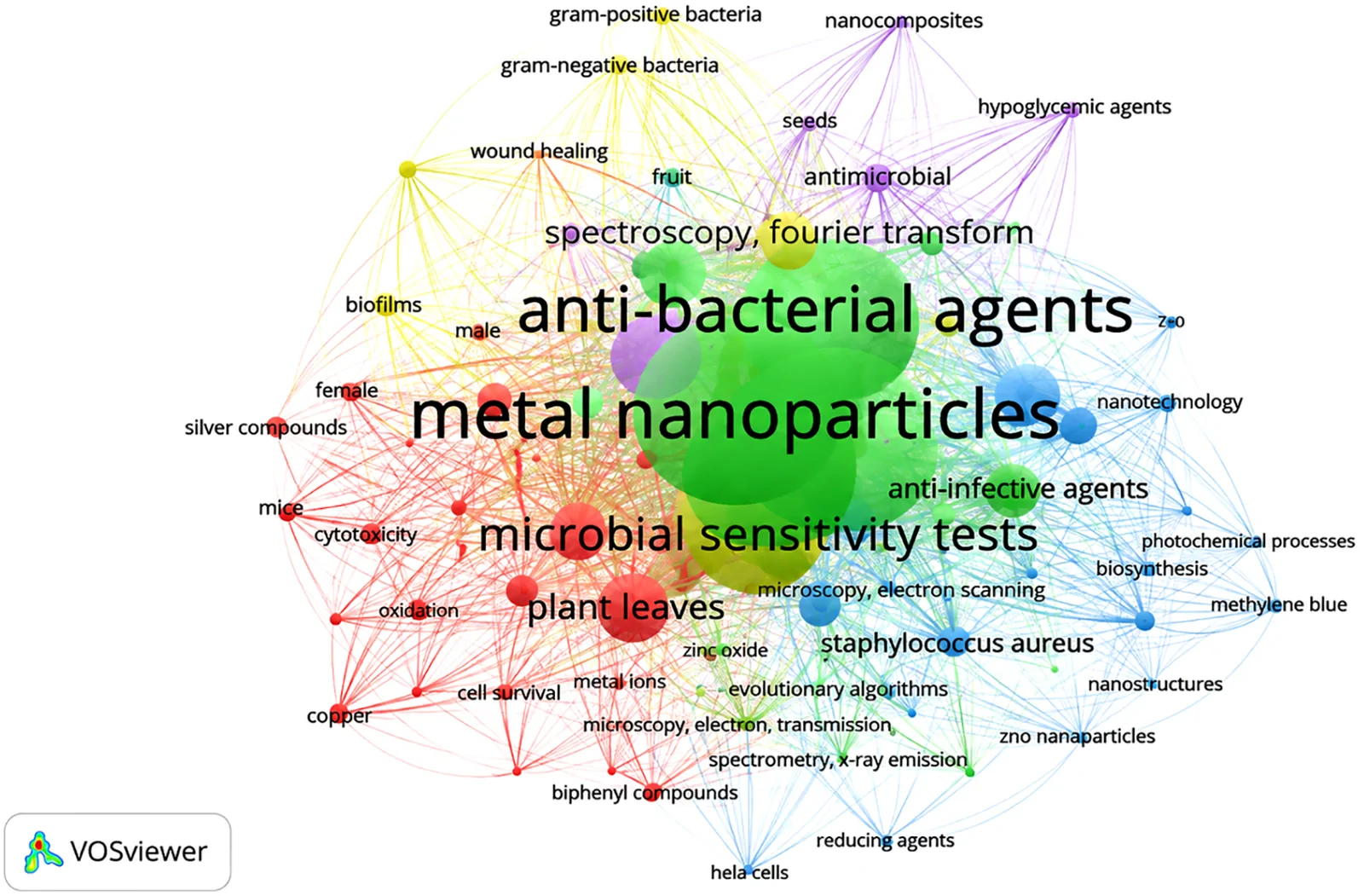
VOS viewer-based keyword co-occurrence map showing research trends in PNPs and antimicrobial applications.

The first red group was principally related to green synthesis and plant-based nanoparticles synthesis. This cluster highlights the growing emphasis on plant-mediated and so-called green nanoparticle synthesis, although the environmental sustainability of these approaches requires assessment of their entire life cycle. The third cluster focused on experimental characterization and microbial interactions, including *E. coli, Staphylococcus*, as well as spectroscopic techniques. This means important antimicrobial mechanisms and evidence of nanoparticle effectiveness from lab-studies. The fourth cluster illustrates biofilm Inhibition and more sophisticated antimicrobial applications, with keywords such as biofilm and antimicrobial activity; in another words, PNPs play an important role in both controlling biofilm associated infections and increasing antimicrobial activity. On the other hand, food associated keywords play a less prominent role; suggesting that food-based use of PNPs remain a new and underexplored topic, indicating a research gap that needs to be addressed (Barciela et al., 2023; Kaushal et al., 2024).

## 3 Food safety and food preservation: a major concern

### 3.1 Major antimicrobial foodborne pathogens and their transmission

The increasing ability to resist conventional antibiotics and the spread of antimicrobial-resistant foodborne pathogens through contaminated food make them a serious public health concern. Common pathogens, for instance, such as *Salmonella* spp.*, Escherichia coli, Listeria monocytogenes, Staphylococcus aureus, and Campylobacter jejuni*, cause various foodborne diseases. Foodborne pathogens enter the food chain at various stages, including primary production, processing, handling, storage, and distribution (Farrukh et al., 2025). A number of meat and poultry products, eggs and dairy, and various fresh fruits and vegetables can act as reservoirs for these pathogens. In some cases, microorganisms can survive in a number of different environments, and some can even form biofilms on food and food coming into contact with surfaces. Biofilm formation can enhance microbial persistence in food-processing and food-contact environments and may increase tolerance to antimicrobial stresses (Kabeya et al., 2025). The AMR profiles of foodborne pathogens can be different even between species and strains. Some *Salmonella* can develop multidrug resistance against *β*-lactams, tetracyclines, sulfonamides, and quinolones. In case of *E. coli*, resistant strains can contain multiple resistance genes. *L. monocytogenes* is unique amongst all foodborne pathogens because it can grow and persist in food processing environments and can even develop strong biofilms. Because of these characteristics, antimicrobial-resistant strains and persistent or biofilm-associated populations represent relevant targets for alternative antimicrobial interventions; however, resistance, tolerance and persistence should be considered distinct phenotypes. The presence of any antimicrobial activity should not be considered evidence for mitigating AMR, but rather this should be reserved for the evaluation of other factors. This is particularly true for PNPs because, while they may help reduce contamination and persistence, the impact on AMR and resistance gene dissemination should be examined (Huoy et al., 2025). **Table 2** summarizes important foodborne pathogens as well as their food sources, characteristics of transmission, AMR phenotypes, resistance determinants, and biofilm-associated persistence.

**Table 2:** Major foodborne bacterial pathogens, antimicrobial resistance characteristics, and relevance to phyto-nanoparticle interventions.

Pathogen	Food sources and study context	AMR phenotypes and resistance mechanisms	Resistance genes and quantitative AMR evidence	Biofilm/persistence capability	Relevance to phyto-nanoparticle intervention	Reference
*Salmonella enterica*	Poultry, eggs, meat, milk and fresh produce. Federal District, Brazil; retail chicken meat; 2023	MDR; β-lactam, tetracycline, sulfonamide and quinolone resistance; β-lactamase production, target modification, efflux and mobile resistance elements	*blaTEM, blaCTX-M, sul, tet, qnr*; 53.8% MDR among retail chicken-meat isolates; resistance to amoxicillin 83.3%, sulfonamides 64.1%, and tetracycline 46.2%	Biofilm formation contributes to persistence on food-contact environments	Potential target against resistant *Salmonella*; antibacterial activity should not be interpreted as direct AMR mitigation	(Kabeya et al., 2025)
*Escherichia coli*	Beef, poultry, pork, leafy vegetables, sprouts and unpasteurized milk. North Carolina, USA; retail chicken, turkey and pork; 2022	MDR involving β-lactams, tetracyclines, aminoglycosides and quinolones; β-lactamases, efflux, target modification and plasmid-mediated resistance	*tetB, tetA, aph(3’’)-Ib, blaTEM-1A, qnrB19, sul1*; AMR genes detected in 77.8% (91/117) isolates; 35.9% (42/117) were MDR	Biofilm formation can enhance persistence and antimicrobial tolerance	Potential target for resistant *E. coli*; requires strain- and food-matrix-specific validation	(Aworh et al., 2024)
*Listeria monocytogenes*	Ready-to-eat meat, dairy products and seafood. South Africa; ready-to-eat food study; 2023	Resistance to sulfonamides, trimethoprim, β-lactams, macrolides and tetracyclines; acquired resistance determinants and adaptive tolerance associated with food-processing persistence	Resistance genes vary among isolates; resistance rates among 194 isolates were 61.86% for sulfamethoxazole, 56.19% for trimethoprim, 42.27% for amoxicillin and 41.24% for penicillin	Strong biofilm formation and environmental persistence	Relevant target for phyto-NP-based food-contact interventions; resistant/persistent strains require direct validation	(Aworh et al., 2024)
*Staphylococcus aureus*	Milk, dairy, meat, poultry, salads and ready-to-eat foods. Mansoura, Egypt; retail animal-origin foods; 2022	MRSA; MDR; β-lactam and macrolide resistance; altered penicillin-binding protein, β-lactamase production and acquired resistance mechanisms	*mecA, blaZ*; detected in 32.69% (85/260) of food samples; MRSA 11.15% (29/260); 89.4% of isolates showed a MAR phenotype; *mecA* and *blaZ* detected in 34.11% and 82.35%, respectively	Biofilm formation contributes to persistence and reduced antimicrobial susceptibility	Potential target against MRSA/MDR *S. aureus*; food-system validation is required	(Ghabbour et al., 2022
*Campylobacter jejuni*	Poultry, raw milk, meat and contaminated water. USA; broilers and retail chicken; 2022	Fluoroquinolone/quinolone and tetracycline resistance; MDR; target-site mutation and efflux	*gyrA, tet(O)*; 13.6% MDR; nalidixic-acid resistance 49.2%; tetracycline resistance 23.7%; *gyrA* detected in 52.4% and *tet(O)* in 14.6%	Biofilm formation contributes to environmental persistence	Potential application in poultry/food systems; efficacy against resistant strains requires direct validation	(Poudel et al., 2022)
*Vibrio* spp.	Seafood, shrimp, shellfish and contaminated water. Inland saline shrimp-culture environments, India; 2023	Multidrug resistance to β-lactams and other antimicrobial classes; β-lactamases, efflux and acquired resistance determinants	Strain- and species-dependent; high resistance to several antibiotics reported among food-associated *Vibrio* isolates, with values varying according to species and antimicrobial tested	Biofilm formation contributes to persistence in aquatic environments	Potential relevance to seafood/water sanitation; direct AMR-mitigation evidence remains limited	(Sudan et al., 2023)
*Bacillus cereus* group	Rice, cereals, flour, pasta, dairy, meat and ready-to-eat foods. Southern China; food surveillance; 2020–2023	β-lactam resistance; MDR; β-lactamase-mediated resistance and acquired resistance determinants	*bla1, bla2, BcI, BcII, blaTEM-116*; 98.5% resistant to penicillin and 98.9% to ampicillin; 18.4% of sequenced isolates were MDR	Spore formation and biofilms contribute to persistence	Relevant to food preservation and surface-control applications; MDR-specific efficacy requires direct testing	(Zheng et al., 2024)
*Shigella* spp.	Vegetable salads, raw vegetables, ground meat, raw milk and contaminated water. Iran; food samples; 2021	MDR involving β-lactams, sulfonamide/trimethoprim and tetracycline resistance; β-lactamase production and acquired/mobile resistance determinants	*blaSHV* and other resistance determinants; food prevalence 4.44% among 405 samples; all recovered isolates were MDR; high resistance to several tested antibiotics	Biofilm formation can contribute to environmental persistence	Potential target against resistant *Shigella*; direct food-system and AMR-related validation remains limited	(Pakbin et al., 2021)

Antimicrobial resistance, tolerance, and persistence are distinct phenotypes. Resistance refers to a measurable reduction in susceptibility to an antimicrobial agent, whereas tolerance describes the ability of microorganisms to survive antimicrobial exposure without necessarily exhibiting an increased MIC. Persistence refers to the survival of a phenotypically tolerant subpopulation during antimicrobial stress. Biofilm formation may promote persistence and antimicrobial tolerance but is not synonymous with antimicrobial resistance.

### 3.2 Mechanism of antimicrobial resistance in foodborne pathogens

Through various molecular and biochemical mechanisms conferring antibiotic resistance, these AMR foodborne pathogens survive and persist in the food system (Blair et al., 2015). One of the most common mechanisms, known as enzymatic degradation, involves pathogens producing enzymes such as beta-lactamases that inactivate antibiotics. In addition, another mechanism is efflux pump systems, which actively remove antimicrobial agents from bacterial cells, thereby minimizing the intracellular drug concentrations (Fernández and Hancock, 2012). At the target site, mutations in ribosomal proteins/enzymes further diminished the antibiotic binding affinity even more.

Another well documented mode of action involving reduce membrane permeability is the alternation of porin, to slow antibiotics penetration into bacterial cells. The traits could be transmitted rapidly from the parent to offspring in via Horizontal gene transfer (HGT). HGT also occurs through conjugation, transformation, and transduction (Thomas and Nielsen, 2005). In the rapid dissemination of antimicrobial resistance genes, mobile genetic elements, such as plasmids, integrons, and transposons, play a key role. The genetic exchange that facilitates the spread of resistance across different bacterial species within the food system is illustrated in **Figure 4**. The formation of biofilms by foodborne pathogens plays an important role in the development of antimicrobial resistance. Biofilms consist of microbial communities that are encased in a self-produced extracellular polymeric substance (EPS) matrix. This matrix allows the community to adhere to both biotic and abiotic surfaces. Biofilms survive in food processing equipment (e.g. Processing equipments, pipelines), on contact surfaces and in food packaging systems and pose a threat to food quality and safety. The EPS matrix of the biofilm acts as a physical barrier, preventing penetration of antibacterial agents and thus inhibiting their activity. It also promotes low metabolic rate and growth rates in bacteria, thereby increasing tolerance to the antibiotic compounds. Moreover, horizontal transfer of antimicrobial resistance genes occurs easily in biofilms and could turn some non-pathogenic bacteria turn into a new pathogens with antimicrobial resistance properties. It has been shown that *L. monocytogenes* and *S. aureus* readily form biofilms and therefore are more likely to persist in the food-processing environment (Dawan et al., 2022). Thus, the biofilm exhibits resistance to bacteria, resulting in recurring contamination of the food system, and expanding the scope of antimicrobial resistance. So, the mechanism of antimicrobial resistance disruption in biofilms is shown in **Figure 5**.

**Figure 4:**
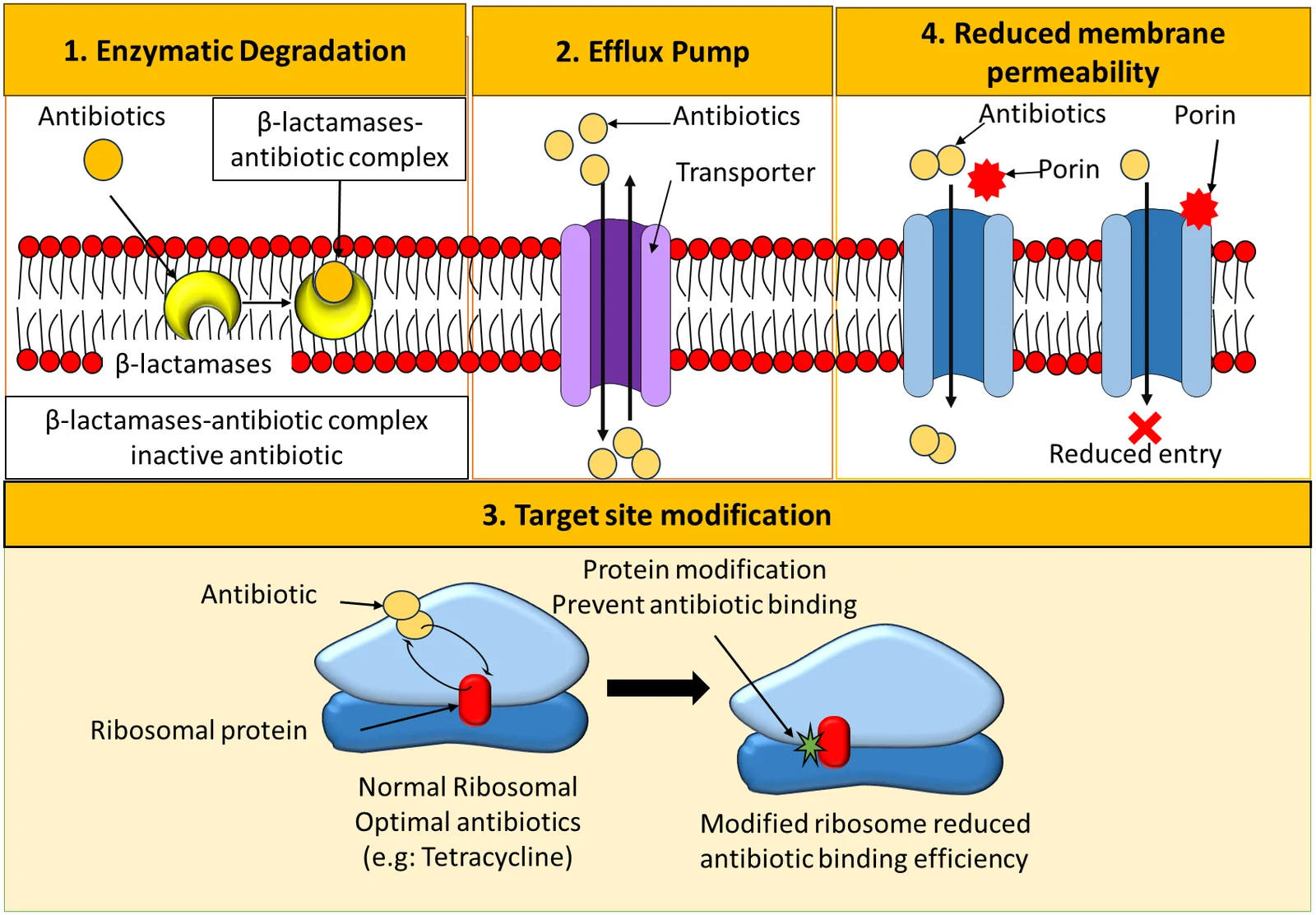
Comprehensive illustration of all four distinct areas: (1) enzymatic degradation, (2) efflux pump, (3) target site modification, and (4) reduced membrane permeability.

**Figure 5:**
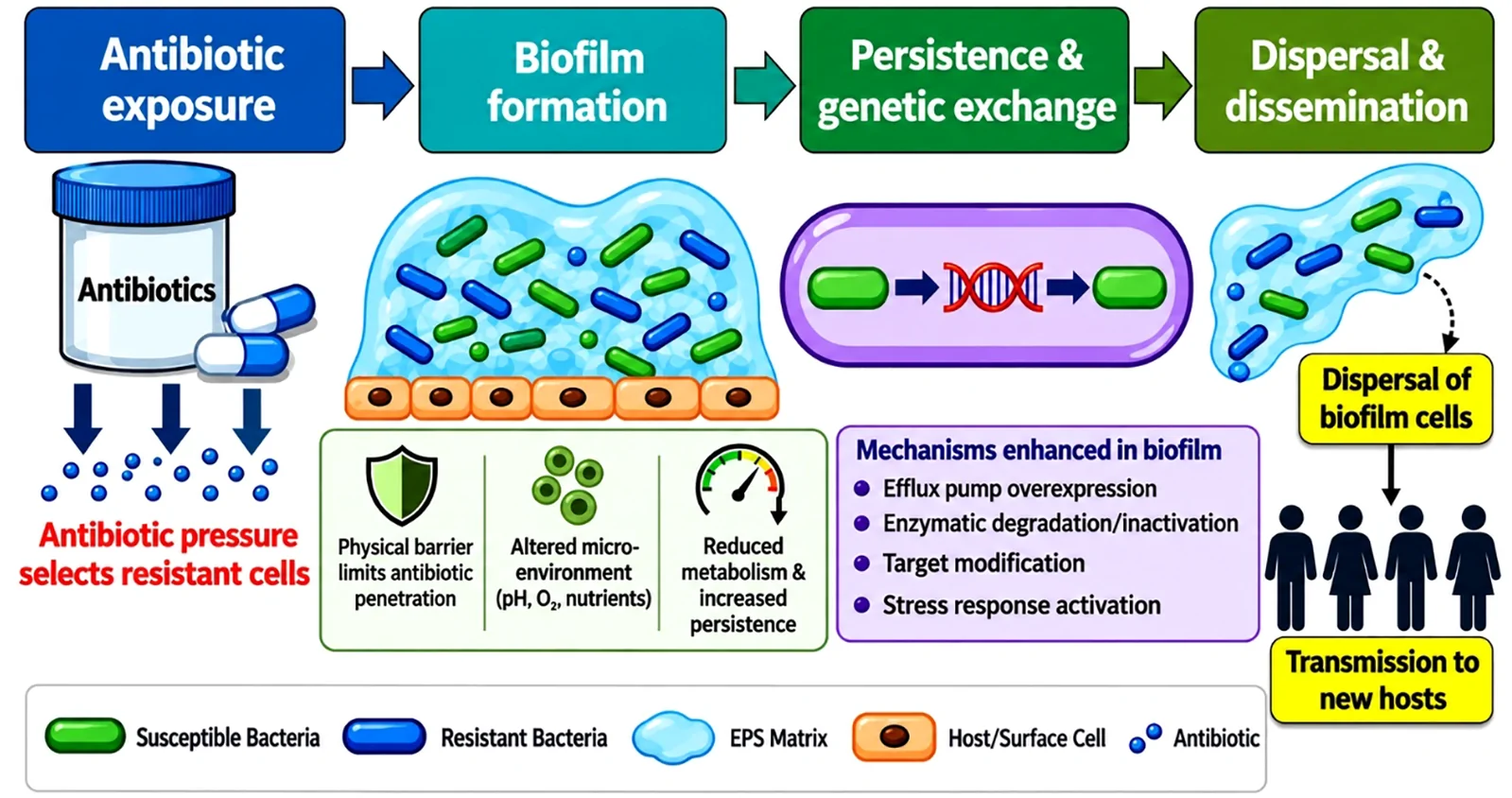
Biofilm-mediated mechanism in antimicrobial resistance.

### 3.3 Public health, environment, and global impact of AMR in the food system

AMR represents a complex global challenge in the food system that extends beyond human health, encompassing interactions among animals, the environment, and ecosystems within the One Health framework. In this framework, the food chain acts as a critical interface, facilitating the transmission of AMR among animal, human, and environmental reservoirs. Different environmental matrices, including soil, water, and agricultural runoff, play a significant role in regulating AMR genes, thereby contributing to their persistence and dissemination within the food system (Kanojiya et al., 2026). The resistome refers to the entire population of AMR genes present in a community, highlighting the complexity and widespread nature of resistance in food-associated ecosystems. The increased resistance of multidrug-resistant pathogens significantly limits available treatment options, resulting in prolonged illness, increased hospitalization rates, and higher mortality rates.

Along with this, AMR also contributes to the economic burden by increasing health care costs, minimizing productivity, and affecting the global supply chain. Driven by globalization, food trade further accelerates the spread of AMR across borders, introducing it into different geographical areas. Major foodborne pathogenic microorganisms are summarized in **Table 3**.

**Table 3:** Major Foodborne pathogenic microorganisms, diseases, and food items associated with these bacteria (Source: Compiled and adapted from CDC (2025a, b).

Pathogenic microorganism	Major food sources	Disease manifestation	Incubation period	Transmission route	Severity/outcome	High-risk groups	Global burden/outbreak relevance
*Salmonella enterica* (non-typhoidal *Salmonella*)	Raw/undercooked poultry and meat, eggs, unpasteurized milk/juice, raw fruits and vegetables	Salmonellosis: diarrhea, fever, abdominal cramps, vomiting; occasionally invasive bloodstream infection	6 h–6 days, usually 6–48 h	Ingestion of contaminated food/water; fecal–oral transmission; animal contact	Usually self-limiting gastroenteritis; invasive disease can occur	Young children, older adults, and immunocompromised people	A major global foodborne pathogen and an important cause of outbreaks
*Escherichia coli* (STEC, including O157:H7)	Undercooked ground beef, unpasteurized milk/juice, leafy vegetables, raw sprouts, contaminated water	Diarrhea, often bloody; severe abdominal cramps; hemolytic uremic syndrome (HUS) may develop	3–4 days	Fecal–oral transmission through contaminated food/water; animal and person-to-person transmission	Usually, diarrheal illness; approximately 5–10% of diagnosed *E. coli* infections may lead to HUS according to the CDC	Children <5 years and adults ≥65 years are at increased risk of severe disease.	An important cause of foodborne disease and outbreaks worldwide
*Listeria monocytogenes*	Ready-to-eat meats/deli meats, soft cheeses, raw/unpasteurized milk, smoked fish, and some raw produce	Listeriosis; invasive disease can cause sepsis, meningitis/meningoencephalitis; pregnancy-associated infection can cause fetal loss or neonatal disease	Usually within 2 weeks; may occur much later in invasive disease	Primarily foodborne; transplacental transmission during pregnancy	Potentially severe; invasive listeriosis has high hospitalization and mortality; pregnancy-associated infection can cause miscarriage, stillbirth or neonatal disease	Pregnant women, newborns, adults ≥65 years, immunocompromised people	An important foodborne pathogen associated with serious outbreaks and disproportionate mortality
*Staphylococcus aureus*	Foods handled after cooking, sliced meats, sandwiches, salads, dairy products, pastries, and other ready-to-eat foods	Staphylococcal food poisoning: nausea, vomiting, abdominal cramps, and diarrhea caused mainly by preformed enterotoxins	30 min–8 h, usually 2–4 h	Ingestion of food containing preformed enterotoxin, commonly following contamination during food handling	Usually acute and self-limiting; dehydration may occur	All age groups; risk depends mainly on contaminated food exposure	An important cause of food intoxication and foodborne outbreaks
*Campylobacter jejuni*	Raw/undercooked poultry, raw milk, contaminated water, and other animal-derived foods	Campylobacteriosis: diarrhea, often bloody, fever, abdominal pain; nausea/vomiting may occur	2–5 days	Ingestion of contaminated food/water; animal contact	Usually resolves within about 1 week; complications can include reactive arthritis, IBS, and Guillain–Barré syndrome	Adults ≥65 years, pregnant women, and immunocompromised people	One of the major bacterial causes of foodborne gastroenteritis globally
*Vibrio* spp. (including *V. cholerae*)	Raw/undercooked shellfish and seafood; contaminated water	*V. cholerae*: cholera with profuse watery diarrhea; other *Vibrio* spp. can cause gastroenteritis and wound/severe systemic infections	Typically 1–2 days for foodborne Vibrio illness; species-dependent	Consumption of contaminated seafood/water; some species also enter through wounds exposed to contaminated water	Can range from mild gastroenteritis to severe dehydration; cholera can cause life-threatening dehydration and shock	People consuming raw/undercooked seafood; risk varies by species and underlying health status	Important water- and seafood-associated foodborne pathogen; cholera remains a major global public-health concern
*Bacillus cereus*	Cooked/reheated rice, cereals, pasta, starchy foods, dairy, and other prepared foods	Emetic syndrome: nausea/vomiting; diarrheal syndrome: diarrhea and abdominal cramps	1–6 h for emetic syndrome; 6–24 h for diarrheal syndrome	Ingestion of preformed toxin or viable cells/spores in contaminated food	Usually mild and self-limiting; severe disease is uncommon	All age groups; severity depends on exposure and host factors	Common food-poisoning organism associated particularly with improperly stored prepared foods
*Shigella* spp.	Contaminated salads/vegetables, ready-to-eat foods, contaminated water, and foods handled by infected persons	Shigellosis: diarrhea, fever, abdominal pain; diarrhea may be bloody; tenesmus may occur	Usually 1–2 days	Fecal–oral transmission, including contaminated food/water and person-to-person spread	Usually self-limiting, but can cause prolonged/severe diarrhea and complications.	Young children, older adults, and immunocompromised people	Major foodborne and enteric pathogen with recurrent outbreaks and substantial global burden
*Clostridium botulinum*	Improperly canned, preserved, or fermented foods; certain homemade preserved foods	Foodborne botulism: blurred/double vision, difficulty swallowing, muscle weakness, and descending flaccid paralysis	Usually 18–36 h	Ingestion of preformed botulinum neurotoxin in improperly processed food	Rare but potentially fatal neurological disease; respiratory failure can occur	People consuming improperly canned, preserved, or fermented foods	Rare but severe foodborne disease; outbreaks can occur following consumption of contaminated preserved foods

### 3.4 Limitations of the current antimicrobial strategies in the food system

The rapid emergence and adaptation of resistant microorganisms, along with the proven ineffectiveness of conventional antimicrobial strategies, chemical preservatives, and antibiotics. Besides that, the rapid use of antibiotics and animal husbandry also significantly contribute to the development of multidrug-resistant (MDR) pathogens, thereby reducing the effectiveness of existing therapeutic options. Although chemical preservatives are used in large quantities, their effectiveness against biofilm-forming pathogens is very limited. Although the natural products, such as plant extracts, essential oils, and bacteriocins, have shown potential, their poor stability, low bioavailability, and rapid degradation limit their application. The use of synthetic preservatives is increasingly influenced by consumer demand for minimally processed foods with fewer synthetic additives. In food systems, limitations stemming from toxicity concerns, regulatory requirements, and challenges in large-scale production may also hinder the development and advancement of emerging antimicrobial technologies (Kanojiya et al., 2026). These limitations necessitate an urgent need for alternative and sustainable antimicrobial strategies to combat AMR in the food system. It is established that sophisticated processes govern the AMR system, including adaptation, biofilm formation, environmental spread, and global transmission through the global food chain. These strict regulations make it more challenging as, the limitations that exist in current antimicrobial strategies further compound the problem and demand new approaches to overcome the problem. Accordingly, phyto-nanotechnology has gained considerable attention as an antimicrobial strategy to control foodborne pathogens and improve food safety. Its contribution to AMR mitigation is most relevant when it targets antibiotic-resistant pathogens or limits the emergence and dissemination of antimicrobial resistance, rather than focusing solely on antimicrobial activity (Alem et al., 2025).

## 4 Green engineering of PNPs

### 4.1 Plant-mediated synthesis and role of phytochemicals

Plant-mediated green synthesis is used to produce metallic and metal-oxide PNPs, in which plant extracts serve as natural reducing, stabilizing, and capping agents. To reduce metal ions to nanoparticles, plant-derived biomolecules such as flavonoids, phenolic compounds, terpenoids, and proteins play a critical role. The phytochemicals present in the plant extract not only facilitate the reduction of metal ions but also act as capping agents, stabilizing the nanoparticles and preventing aggregation. In contrast to some traditional chemical synthesis pathways, plant-assisted synthesis can minimize the need for synthetic reducing and stabilizing agents. Still, ultimately, its environmental impact would depend on several parameters, such as plant growth, use of solvents, energy, and water, the need for purification, waste generation, and the environmental impacts of the resulting nanoparticles. Silver nanoparticles, gold nanoparticles, and zinc oxide and copper oxide nanoparticles were synthesized using different parts of the plant, i.e., stem, flower, leaves, root, and fruit. The physical dimensions (shape and size) and characteristic traits of PNPs were influenced by pH, temperature, reaction time, and the amount of plant extract used. To design an effective and functional nanoparticle, these parameters must be considered and controlled. In PNPs, the physicochemical properties and biological activities are influenced by interactions between plant-derived phytochemicals and metal ions, leading to the formation of nanoparticles with unique surface functionalization (Chowdhury et al., 2020). The presence of functional groups, such as hydroxyl, carbonyl, and amine groups, on the surface of nanoparticles increases their stability. It facilitates interactions with microbial cells at the nano-bio interface. Moreover, targeting the specific antimicrobial system can be achieved by developing precise control over nanoparticle characteristics, such as size, morphology, and surface modifications, through optimized synthesis parameters. Some benefits also accrue however, the present reports on PNP synthesis are used with caution because the variability among the synthetic methodologies complicates reproducibility and comparison of the results across reports. This variability is evident from recent original studies, for instance, AgNPs synthesis using *Syzygium polyanthum* leaf extract shows a zone of Inhibition around 10.16-13.16mm, MIC values of 8–125 ug/mL, and MBC values 8–250 ug/mL against gram-positive and gram-negative foodborne pathogens. Conversely, the *Agaricus bisporus*-mediated AgNPs display a large zone of Inhibition of 20 to 48 mm against selected foodborne bacteria and also show good cytocompatibility, indicating that antimicrobial activity varies with the properties of the nanoparticles and their biological source. Similarly, AgNPs prepared from *Punica granatum* peel exhibit activity against foodborne bacterial and fungal pathogens; however, effectiveness varies among pathogens, despite using the same synthesis method. As such, the above-mentioned observations indicate significant variation in antimicrobial efficacy reported across different systems of PNPs, owing to differences in botanical origin, phytochemical composition, size, shape, surface properties, synthetic procedures, microorganisms studied, and the methodology used for antimicrobial activity testing. The above-mentioned variations can lead to apparent contradictions that may arise not only from actual discrepancies in nanoparticle activity but also from different methodologies used in studies (Mohanta et al., 2017; Chanda et al., 2019; Khan et al., 2019; Geremew et al., 2022; Hayat et al., 2022; Farouk et al., 2023).

### 4.2 Standardization, characterization, and mechanistic challenges of PNPs

Despite the widespread acceptance of plant-mediated synthesis, often proposed as an environmentally friendly technique compared with classical chemistry, the process of nanoparticle production itself is far from standard or replicable. In contrast to chemical reduction agents, plant extract is made up of a mixture of different kinds of secondary metabolites, for example, flavonoids, phenolic acids, tannins, terpenoids, alkaloids, proteins, polysaccharides, and more, which act as reducing, stabilizing, and capping agents. The composition of these secondary metabolites varies substantially depending on the plant, plant part, developmental phase, geographical location, season, and other factors. Thus, even with the same synthesis protocol, variations in the composition of the phytochemicals may result in nanoparticles that differ considerably in size, shape, crystallinity, surface chemistry, colloidal stability, kinetics of metal-ion release, and antimicrobial activity. This biological variability is one of the main hurdles in reproducibility, comparison of results from published work, and large-scale production (Shakeel et al., 2025). Thus, standardization of plant-extraction procedures, thorough physicochemical characterization, and detailed documentation of the synthetic parameters are imperative for reproducible nanoparticle production and for understanding the antimicrobial efficacy summarized in **Table 4**.

**Table 4:** Systematize phytochemical characterization of PNPs for antimicrobial application.

Characterization category	Techniques	Information obtained	Scientific significance for antimicrobial interpretation	Typical instrument	Critical parameters to report	Reference
Optical characterization	UV–Visible spectroscopy (UV–Vis)	Surface plasmon resonance (SPR), nanoparticle formation, and reaction kinetics	Confirms successful nanoparticle synthesis, evaluates reaction completion, and monitors nanoparticle stability during storage	UV–Vis spectrophotometer	SPR wavelength (λmax), absorbance intensity, peak width, reaction time	(Tippayawat et al., 2016)
Surface chemistry	Fourier-transform infrared spectroscopy (FTIR)	Functional groups, phytochemical capping molecules, metal–ligand interactions	Identifies biomolecules responsible for nanoparticle reduction, stabilization, and potential antimicrobial activity	FTIR spectrometer	Wavenumber (cm^-1^), peak assignment, functional group identification	(Iravani, 2011)
Crystal structure	X-ray diffraction (XRD)	Crystal phase, crystallinity, crystallite size, phase purity	Confirms nanoparticle crystalline structure, which influences ion release, ROS generation, and antimicrobial activity	X-ray diffractometer	2θ values, Miller indices, crystallite size (Scherrer equation), phase identification	(Husen and Siddiqi, 2014)
Morphological characterization	Transmission electron microscopy (TEM)	Particle size, morphology, lattice fringes, size distribution	Provides accurate nanoparticle dimensions and morphology, which strongly influence bacterial interaction and cellular uptake	Transmission Electron Microscope	Mean particle size, particle shape, lattice spacing, size distribution	(Schröder et al., 2013)
Surface morphology	Scanning electron microscopy (SEM)	Surface morphology, aggregation state, particle dispersion	Evaluates nanoparticle aggregation and surface architecture affecting antimicrobial performance	Scanning Electron Microscope	Surface morphology, aggregation, particle distribution	(Slavin et al., 2017)
Colloidal stability	Dynamic light scattering (DLS)	Hydrodynamic particle size and size distribution	Determines nanoparticle dispersion behavior in aqueous media and predicts biological interactions	Dynamic Light Scattering analyzer	Hydrodynamic diameter, PDI	(Loo et al., 2018)

Physicochemical characterization alone is insufficient to identify the mechanism by which the nanoparticles exert their antibacterial effect, because the observed physiological effects may result from any combination of compounds used in nanoparticle synthesis. Plant-mediated nanoparticles have antibacterial effects from the nanoparticle core, metal ion dissolution (e.g., Ag^+^, Zn^2+^, or Cu^2+^), a phytochemical capping agent, leftover plant extract, or a metal precursor salt, each contributing singly or synergistically. Hence, suitable control studies are needed to separate the individual contributions of each component. They would comprise a plant extract-alone control, a metal salts-alone control, a purified nanoparticle suspension control, and a measurement of metal ions in solution by ICP–MS or ICP-OES. In addition, chromatographic studies such as HPLC or LC-MS would help identify any phytochemicals present.

In contrast, dialysis, ultrafiltration, and chelation studies would help distinguish the effect of nanoparticles from that of ions on antimicrobial activity. Failure to conduct such control studies would result in misinterpretations of the antimicrobial mechanism. Physical and chemical characterization of the NPs is also important for characterizing the impact of anti-biofilm properties. This importance stems from the interaction between intrinsic physicochemical properties of nanoparticles, and the structure of microbial biofilms. Biofilms represent the major mode of bacterial lifestyle, structured a three‐dimensional microbial consortium entrapped into the biofilm matrix consisting of extracellular polymeric substance (EPS; proteins, polysaccharides, lipid, or extracellular DNA), therefore this provides a complex microenvironment and excellent resistance to antimicrobial substances. Furthermore, nutrients, pH, and oxygen gradients develop within the matrix of mature biofilms contributing to a mixture of microbial populations with distinct metabolic activities, each with varying responses to antimicrobial agents, with some cells acting as persister cells. Consequently, nanoparticle diffusion, metal ion leaching, and interactions among phytochemicals within biofilms differ markedly from those in traditional planktonic antimicrobial systems. It is thus important to understand the association between physical and chemical properties of nanoparticles and biofilm formation to develop effective PNPs-based antimicrobials.

Although PNPs have shown potential in combating biofilms, full biofilm removal remains critical owing to the structural and physiological heterogeneity characteristic of mature biofilms. While nanoparticles can diffuse through the outer layers of the biofilm, diffusion into deeper layers is limited by the EPS layer, making persister cells less accessible to nanoparticles. Therefore, many published scientific studies have demonstrated a reduction in biofilm biomass, not eradication of the biofilm itself, and any remaining bacteria can recolonize the surfaces. Hence, future studies should focus on increasing nanoparticle penetration through strategies such as surface modifications, enzyme-induced EPS breakdown, stimuli-sensitive or controlled-release carriers, and combination therapies that include existing antimicrobial agents or enzymes capable of degrading the biofilm matrix. In addition, there should be incorporation of three-dimensional biofilm models, confocal laser scanning microscopy, live-dead staining, and quantification of biofilm penetration. However, these findings should be evaluated critically because most studies rely on short-term *in vitro* assays that employ different synthesis protocols, nanoparticle characterization methods, microbial strains, and antimicrobial susceptibility tests, thereby limiting direct comparison and extrapolation to practical food systems or *in vivo* conditions. Physicochemical characterization alone cannot establish the antimicrobial activity of the nanoparticle-mediated system; therefore, experimental strategies must be used to distinguish nanoparticle-specific effects from residual phytochemicals, dissolved metal ions, and unreacted precursors.

Recent mechanistic reports indicate that diverse phytochemicals are involved in nanoparticle formation and that a minimal quantity of capping agents would allow the remnants of the bound phytochemicals to be responsible for antimicrobial activity if not handled precisely. Furthermore, the effect of physicochemical properties on antibacterial activity must be addressed in light of release of Ag+ ion release, as *in vitro* dissolution can confer to antibacterial activity. Hence, carefully controlled experiments and purifications may lead to discovery of the causative factor linking Physicochemical properties and antibacterial activity of AgNPs. These can be revealed by control study with purified nanoparticles, plant extract, metal ion, metal salt precursors solution, control study without any treatment etc. accompanied by estimations of dissolved metal, Surface chemistry by FTIR and XPS, etc., since there exists methodology to distinguish, antimicrobial activity induced by nanoparticles core, metal ion, residue phytochemicals, plant extract and precursor’s.

### 4.3 Mechanism of antimicrobial action by PNPs

The metallic and metal oxide PNPs exhibit antimicrobial activity through multiple synergistic mechanisms that target microbial cells at the structural, biochemical, and genetic levels. The structural relationship, quantitative antimicrobial efficacy, and mechanistic evidence of representative PNPs against foodborne pathogens are summarized in **Table 5**. The production of reactive oxygen species (ROS), including superoxide radicals, hydroxyl radicals, and hydrogen peroxide, is a primary mechanism inducing oxidative stress in microbial cells. The generation of higher ROS levels has been linked to the oxidation of lipids, proteins, and DNA in some nanoparticle studies. Nonetheless, ROS should not automatically be considered the primary agent unless there is quantifiable evidence to that effect, since antimicrobial action may also result from membrane damage and metal-ion toxicity. The PNPs, which can also bind to microbial cell membranes, ultimately disrupt membrane structure and increase permeability, causing leakage of intercellular materials. The electrostatic interaction between the positively charged nanoparticle and the negatively charged bacterial cell surface increases attachment and facilitates membrane destabilization. In addition, nanoparticles can penetrate microbial cells and interfere with intercellular processes, including enzyme activity and metabolic pathways. They can also bind to nucleic acids, leading to disruption of the DNA replication and translation process. They are another mechanism that inhibits quorum sensing, thereby disrupting microbial communication and reducing virulence factor production. Although PNPs exhibit multiple antimicrobial mechanisms, most mechanistic evidence comes from *in vitro* studies under simplified laboratory conditions. Variations in nanoparticle composition, phytochemical capping, particle size, and experimental protocols limit direct comparison among studies, while validation in complex food matrices and *in vivo* models remains limited. Therefore, the relative contribution of individual antimicrobial mechanisms requires further standardized investigation (Geremew et al., 2022; Hayat et al., 2022; Fayed et al., 2025).

**Table 5:** Structure–activity relationship, quantitative antimicrobial efficacy, and mechanistic evidence of representative phyto-nanoparticles against foodborne pathogens.

PNP composition	Plant source & synthesis	Physicochemical characteristics	Foodborne pathogens	Antimicrobial testing & efficacy	Mechanism & ion contribution	Key limitations	Reference
AgNPs	*C. sinensis* (Pu-erh tea); AgNO_3_ reduced by aqueous extract; phytochemicals acted as reducers/capping agents	4.1 nm; spherical; polyphenols/proteins (FTIR); PDI/zeta: NR	*E. coli*, *S. typhimurium*, *S. enteritidis*	Broth microdilution, MIC/MBC, time-kill; MIC/MBC: 3.9–7.8 µg/mL; 0.5–8× MIC, ≤24 h	Membrane damage, oxidative stress, Ag^+^ release; supported by time-kill, TEM and MIC/MBC; Ag^+^ contribution not quantified	PDI, zeta potential and Ag^+^ release kinetics not reported	(Loo et al., 2018)
ZnO NPs	*T. pratense* flower extract; zinc precursor stabilized by phytochemicals and calcined	20–40 nm; hexagonal/spherical; phenolics/flavonoids (FTIR); PDI/zeta: NR	*E. coli*, *S. aureus*	Broth microdilution, agar diffusion; MIC/MBC and exposure: NR	ROS, Zn^2+^ release and membrane damage; supported by ROS, SEM/TEM and viability assays	Activity varies with size, crystallinity and illumination; testing not standardized	(Matsumoto et al., 2017)
CuO NPs	*M. acuminata* peel extract; copper precursor reduced using aqueous extract	30–60 nm; spherical; polyphenols/proteins (FTIR); PDI/zeta: NR	*E. coli*, *L. monocytogenes*	Broth microdilution, agar diffusion; MIC reported; exposure: NR	Cu^2+^ release, oxidative stress and membrane damage; supported by ROS, permeability and SEM; Cu^2+^ contributes substantially	Greater Cu^2+^ release may increase efficacy and cytotoxicity	(Orji et al., 2018)
AuNPs	*H. rosa-sinensis* leaf extract; phytochemicals reduced chloroauric acid	15–40 nm; spherical; polyphenols (FTIR); PDI/zeta: NR	*E. coli*, *S. aureus*	Agar diffusion and broth dilution; lower activity than AgNPs; exposure: NR	Membrane interaction and phytochemical effects; supported by TEM/membrane integrity; ion release minimal	Activity depends strongly on surface functionalization	(Moaienla et al., 2009)
Fe_3_O_4_ NPs	*E. globulus* leaf extract; Fe^2+^/Fe³^+^ salts co-precipitated with extract	20–30 nm; nearly spherical; zeta −22.4 mV; polyphenols/flavonoids/hydroxyl groups (FTIR); PDI: NR	*E. coli*, *S. aureus*	Agar well diffusion; concentration-dependent inhibition; 25–100 µg/mL, 24 h; MIC: NR	ROS, membrane damage and oxidative stress; supported by SEM/FTIR; Fe ion release not quantified	MIC/MBC, PDI and ion-release data not reported	(Azizi et al., 2013)
MgO NPs	*M. indica* leaf extract; magnesium nitrate stabilized by extract and calcined	25–45 nm; nearly spherical; zeta −18 to −25 mV; polyphenols/flavonoids/hydroxyl groups (FTIR); PDI: NR	*E. coli*, *S. aureus*	Broth microdilution, agar diffusion; concentration-dependent inhibition; MIC reported in some studies; 25–200 µg/mL, 18–24 h	ROS, membrane damage and alkaline surface effects; supported by SEM, ROS and viability assays; Mg^2+^ contribution considered minor	Standardized MIC/MBC and ion-release data limited	(Raj et al., 2025)

Mechanistic evidence was classified as **direct** when supported by experimental techniques such as ROS quantification (e.g., DCFH-DA assay), TEM or SEM imaging, ICP-MS analysis of metal-ion release, RT-qPCR, transcriptomic, or proteomic analyses. **Indirect** evidence refers to conclusions inferred primarily from antimicrobial activity, growth inhibition, or morphological observations without direct mechanistic validation.

The antimicrobial activity of PNPs results from both ion-mediated and particle-mediated mechanisms. These particles release the metal ion, for example, Ag^+^, Zn^2+^, and Cu^2+^, which exhibit the bacterial enzymes, which include oxidative stress, enzymes, and disrupt the intercellular process. On the other hand, intact nanoparticles directly interact with bacterial membranes, causing membrane damage, ROS generation, intercellular disruption, etc. Ion-mediated activity depends upon the rate of metal-ion dissolution; in contrast, particle-mediated activity is strongly influenced by the nanoparticle size. In contrast to that, nanoparticle properties, for example, particle size, which typically varies from 4–100 nm for the green-synthesized AgNPs, and surface phytochemical capping influence the membrane interaction and antimicrobial efficiency. So, both mechanisms provide the antimicrobial activity, and their relative importance also depends upon the nanoparticle composition and experimental environment.

The antimicrobial effects of PNPs depend on a balance between the physical and chemical properties of these nanoparticles and their compositions. Nanoparticle size, shape, charge, chemistry, and capping with phytochemicals may alter attachment and interactions with microbes, thereby affecting metal ion release levels and, consequently, the degree of antimicrobial effect. Among all the parameters, size is considered to have a significant impact on antimicrobial efficiency as the small sized nanoparticles provide a larger surface-to- volume ratio, thereby increasing surface area and enabling their faster, more effective engagement with microbes as well as greater reactivity towards microbes due to enhanced production of ROS. Nanoparticle morphology governs cell internalization by cells and binding to the cell membrane. Plant derived phytoconstituents used as capping agents improve the colloidal stability, release profile of metal ions, and also possess themselves antimicrobial potential including phenolics, flavonoids, terpenoids, tannins etc. Therefore, the mechanism of antimicrobial action depends on the synergistic effects of the composition, particle size, structure, surface characteristics, and the phytochemicals coating the nanoparticles. Although the production of reactive oxygen species (ROS), cell membrane destruction, DNA damage, and the dissolution of metal ions have been established as the primary modes of action of the PNPs, the strength of evidence supporting this claim varies greatly across studies. Most of the studies make conclusions regarding the above-listed mechanisms indirectly based on bacterial growth inhibition, cell membrane disruption, or observation of morphological alterations through electron microscopy; very few actually measure intracellular ROS production, monitor metal ion release kinetics, or carry out transcriptomic and proteomic analysis of cellular targets (Konkal et al., 2026). One limitation is the variation in methodologies employed across research publications. Variations in nanoparticle preparation methods, phytochemical composition, particle size, surface chemistry, microbial strains, cultivation media, pH, oxygen levels, and nanoparticle concentrations have often led to inconsistencies in observed antimicrobial effects. Hence, comparison of antimicrobial effectiveness among different PNPs systems is difficult (Geremew et al., 2022; Kaushal et al., 2024).

Quantitative comparisons reveal significant differences in the antimicrobial potential of PNPs, which can be linked to nanoparticle chemistry, physicochemical characteristics, and experimental conditions. For instance, green-synthesized silver nanoparticles (AgNPs) with a mean size of approximately 4.06 nm displayed MIC values of 3.9-7.8 µg/mL towards *Escherichia coli*, *Salmonella typhimurium*, and *Salmonella enteritidis*, which revealed highly potent antimicrobial activity due to the synergistic action of Ag^+^ ion release, ROS generation, and membrane disruption. Meanwhile, antimicrobial activity of plant-based zinc oxide nanoparticles (ZnO NPs), which usually range from 20 to 40 nm, results from the combination of ROS generation, Zn^2+^ ion release, and membrane damage; however, the reported antimicrobial potential of the nanoparticles is extremely variable due to variations in nanoparticle sizes, methods of synthesis, microorganisms, and test conditions. Similar to CuO NPs, oxidation and membrane damage were induced mainly by Cu ion release but in case of antibacterial effects varied with nanoparticle size, dissolution rate and environment. However, AuNPs being biocompatible and stable, show lower antimicrobial activity than AgNPs and most often require a phytochemical coating to be effective against bacteria. Taking these observations into account, it can be concluded that the antibacterial efficacy of nanoparticles depends on chemical composition, size, morphology, surface chemistry, plant-compound coating, the rate of ion release and testing conditions. While smaller particles may offer more surface area for interaction with bacterial cells, surface charge and plant-compound coatings may alter the particle-bacterial attachment and ion-release behavior. This shows that significant variation in results can occur even when using the same metal due to differences in the physicochemical properties of nanoparticles.

The antimicrobial activity of PNPs depends not only on the metallic core but also on the phytochemicals present on their surface, due to green synthesis. Bioactive compounds, such as flavonoids, phenolic acids, tannins, terpenoids, alkaloids, and saponins, act as natural reducing and capping agents, increasing nanoparticle stability, preventing aggregation, and altering particle surface chemistry. These phytochemicals can modulate the antimicrobial activity of the PNPs by increasing membrane permeability, disrupting metabolic pathways, inhibiting quorum sensing, and exacerbating oxidative stress through synergistic effects with metal nanoparticles. As a result, changes in the plant’s metabolite profile usually lead to changes in the nanoparticles’ size, morphology, surface charge, and antimicrobial activity, even when the same metallic precursor is used. Hence, knowledge of the relationship between the phytochemical profile and the nanoparticle’s physicochemical properties is necessary to develop highly efficient PNPs for food safety purposes. Despite extensive efforts to understand the antimicrobial mechanisms of action of PNPs, several controversies remain regarding some of the processes involved. First, there is controversy over whether nanoparticle-specific activities are responsible for antimicrobial activity or, rather, whether the role is played by metal ions liberated from the nanoparticles themselves. In addition, while ROS has been widely cited as the main mode of antimicrobial activity, other studies have shown efficient antimicrobial activity despite very low levels of ROS generation, indicating that other mechanisms, such as membrane destruction, interactions between proteins within the cell, and metal ion toxicity, could also be as effective. Moreover, variation in nanoparticle synthesis, types and concentrations of phytochemicals, nanoparticle sizes, bacteria species used, and antimicrobial tests used leads to conflicts among studies (Hayat et al., 2022).

Differences in dominant antimicrobial mode between different type of NPs (a) Silver nanoparticles: (1) Ag^+^ ion leaching, (2) ROS overproduction, (3) membrane disruption (b) ZnO nanoparticles: (1) ROS produced from photocatalysis, (2) Zn^+2^ dissolution (c) Cu_2_O nanoparticles: oxidative stress, dissolved Cu^+2^ ions, (d) Gold nanoparticles: relatively weak antimicrobial ability and requires coating with phytochemical substances for better bacteria-killing action. The figure shows that the antimicrobial mechanism is material-specific. A critical mechanistic controversy is whether the antimicrobial effect of PNPs is primarily achieved through the release of dissolved metal ions or through direct particle action. The silver nanoparticles slowly release Ag^+^ ions, while ZnO and CuO nanoparticles release Zn^2+^ and Cu^2+^ ions, respectively, all of which have inherent antimicrobial activity. Consequently, the antimicrobial effect noted above could be due to metal ions dissolved in the solution, particle-cell interactions, or a combination of both, depending on nanoparticle size, dissolution rate, and experimental conditions. Experimental studies have demonstrated that antimicrobial effects can arise from both intact nanoparticles and dissolved metal ions. Using cysteine-capped AgNPs and AgNO_3_ as an ionic silver control, it was shown that toxicity against *E. coli* and *P. aeruginosa* was attributable primarily to Ag^+^ ions rather than to intact nanoparticles, despite nanoparticle dissolution being less than 2% under the tested minimal-medium conditions. Further showed a quantitative relationship between the structural parameters, dissolution, and antimicrobial effects of the AgNPs: in their experiment, up to 2-34% of the total silver is dissolved as ionic silver in equilibrium, with the amount of dissolved silver varying from 0.24 to 4.1 ppm depending on the nanoparticles’ size, shape, and surface treatment; the antimicrobial effect depends on the concentration of the dissolved silver in equilibrium. There is also evidence suggesting that surface-bound phytochemicals can affect antimicrobial efficacy on their own. In a recent study in which AgNPs were synthesized via green chemistry, the phytochemicals isolated from their surface exhibited antibacterial activity against *S. aureus, E. coli*, and *P. aeruginosa*. The MIC values ranged from 62.5 to 1000 μg/mL and were sometimes more effective than the raw plant extracts and the capped or uncapped AgNPs. Such a comparison is important for determining whether the observed effect is specific to the nanoparticles or due to ions released from them, which explains the need for comparison with soluble-salt metal controls and for quantifying released ions. Such a comparative approach is crucial for identifying the primary antimicrobial mechanism of action, especially for nanoparticle design, safety evaluation, and regulation. However, quantitatively separating the effects of the core nanoparticle, ions, and plant phytochemicals effects has not been achieved; therefore, it is best to be cautious in concluding the mechanism.

Findings from numerous studies indicate that preservation of food using PNPs can work. However, the results from these studies cannot be generalized because of several reasons, including, but not limited to, variations in food types and composition, PNP formulations and dosages, environmental conditions, types of microorganisms assessed, and endpoints. It is therefore, prudent to conclude that the benefits reported in these studies are restricted to the systems in which they are assessed. Additionally, insufficient data on migration, exposure, and toxicity of nanoparticles limit the scope of conclusions and understand the practicality of these systems.

### 4.4 Application of PNPs against foodborne pathogens

Metallic and metal oxide PNPs have emerged as effective antimicrobial agents against a wide range of foodborne pathogens, including bacteria and fungi. Resulting from their nanoscale size and high surface area, which enables interaction with microbial cell structures, resulting in improved antimicrobial efficiency. PNPs have demonstrated antimicrobial activity against both Gram-positive and Gram-negative foodborne bacteria, including *Escherichia coli*, *Salmonella* spp., *Listeria monocytogenes*, and *Staphylococcus aureus*. However, antimicrobial efficacy varies considerably among microbial species because differences in cell-envelope architecture, particularly the lipopolysaccharide-rich outer membrane of Gram-negative bacteria and the thick peptidoglycan layer of Gram-positive bacteria, influence nanoparticle penetration, metal-ion diffusion, and susceptibility to oxidative stress. Consequently, the same PNPs formulation may exhibit different levels of activity against different bacterial species. These pathogens are commonly associated with contaminated food products and are major contributors to foodborne disease worldwide. PNPs exert multiple effects through multiple mechanisms, including disruption of cell membranes, leakage of intercellular components, and Inhibition of essential metabolic pathways (Geremew et al., 2022; Khan et al., 2023).

Additionally, ROS generation induces oxidative stress, resulting in protein denaturation, lipid peroxidation, and DNA damage in bacterial cells. The multitarget mode of action of PNPs may reduce the likelihood of resistance development compared with conventional agents/drugs that act via a single molecular target. However, it does not mean that PNPs will be resistance-prone or preemptive and active in evolved resistance. Microorganisms evolve to resist or tolerate nanoparticle-triggered stimuli through diverse physiological and genetic pathways; thus, acquired resistance or tolerance may develop with prolonged or repeated exposure develops.

Apart from antibacterial activity, PNPs also exhibit strong antifungal activity against major food-spoiling fungal genera namely; *Aspergillus*, *Penicillium* as well as *Candida* spp. These fungi are a responsible for food deterioration, mycotoxin synthesis and limited shelf-life of food products (Salem et al., 2022; Tayel et al., 2024; Alghuthaymi, 2025; Hassan et al., 2026).

Fungal cells are degraded by membrane damage to the cell walls or interference with ergosterol biosynthesis, thereby inducing oxidative stress in fungi (Kumari et al., 2019). It can reduce fungal load and the degree of food contamination by inhibiting of fungal spore germination and biofilm formation. PNPs are effective at penetrating existing biofilm and are persistent against the microbial community present in food processing environments. PNPs showed a wide spectrum of activity against Gram-positive and Gram-negative foodborne bacteria. Still, most of the activity is based on laboratory results, and insufficient validation studies have been conducted to assess efficacy against in complex food matrices and under industrial food-processing conditions. Need standard application-oriented studies.

Structural differences in structure among various types of microbes contribute greatly influence sensitivity to PNPs. Gram-negative bacteria have an outer membrane composed of lipopolysaccharides, which prevents the penetration of nanoparticles and the diffusion of released metal ions. Gram-positive bacteria do not possess the above-mentioned outer membrane. Still, they are composed of a thick peptidoglycan wall and similarly, chitin- and glucan-containing cell walls are found in fungi, and viruses and parasitic microorganisms possess an entirely different biological construction and reproduction machinery. In conclusion, activity shown toward bacterial pathogens cannot be extrapolated to that of fungi, viruses and parasitic microorganisms. Thus, in the future, comparative standardization should be carried out using various microorganisms across different microbial groups of microbes and consequently, a definitive antimicrobial spectrum of the plant mediated PNPs could be determined.

#### 4.4.1 Antibacterial activity against antibiotic-resistant pathogens

In recent systematic studies, 3,740 records were screened, and 32 primary studies were included, published between 2020 and 2026, showing that green-synthesized silver nanoparticles exhibited antibacterial activity against various critically important multidrug-resistant pathogens, including MRSA, *E. coli, Pseudomonas aeruginosa, Klebsiella pneumoniae*, and *Acinetobacter baumannii.* AgNPs were spherical, with particle sizes ranging from 4-100nm, and reported MIC and MBC values varied from 2.5 μg/mL to 100 mg/mL and 3.8 μg/mL to 2.5 mg/mL, respectively, reflecting considerable methodological heterogeneity (Girma et al., 2024). Apart from these findings, most evidence was generated under *in vitro* conditions, and a recent exposure to an inhibitor can lead to bacterial adaptation and increased AgNP MICs, indicating that nanoparticles are not immune to the development of resistance (Blair et al., 2015). So, recent evidence supports the antibacterial potential of plant-mediated AgNPs against MDR pathogens; however, it does not yet provide definitive evidence of AMR mitigation.

#### 4.4.2 Reduction of microbial load

The plant-mediated PNPs-based coating and packaging have consistently minimized the microbial populations in refrigerated food systems. Silver nanoparticle-incorporated agar films in beef decreased *Listeria monocytogenes* and *Escherichia coli* O157:H7 by approximately 1 log CFU g^-1^ after 15 days of storage (Hong et al., 2021). Alginate nanoparticles loaded with *Zataria multiflora* and *Cuminum cyminum* essential oils reduced the bacteria counts by 2.0-2.74 log CFU mL^-1^ in the same storage period while preserving sensory quality (Osanloo et al., 2023). The above-mentioned finding shows that microbial control in food matrices; however, because neither study evaluated antibiotic-resistant isolates, antimicrobial resistance genes, or resistance transmission, the reported reduction in microbial load cannot be considered direct evidence of AMR mitigation. They indicate improved food safety, which may indirectly decrease antibiotic demand by reducing foodborne contamination (Zheng et al., 2017; Nayab and Akhtar, 2023; Pandian et al., 2023; Demirbas and Karsli, 2025; Joshi et al., 2025; Trak et al., 2025; Likhar et al., 2026).

#### 4.4.3 Biofilm control

Biofilm inhibition represents a potential mechanism by which plant-derived nanoparticles may reduce the persistence of antimicrobial-tolerant microorganisms. Antibiofilm activity has consistently been demonstrated in the original studies; however, the methodologies and magnitudes vary substantially. For instance, ginger-mediated AgNPs reduced established enterococcal biofilm to 39.14% of the untreated control and decreased bacterial adhesion on urinary catheters to 42.73%. In contrast, chemically synthesized AgNPs reduced biofilm to 65.32% and catheter adhesion to 69.84% under the identical conditions, highlighting the influence of nanoparticle composition on efficacy (Swidan et al., 2022). The AgNPs, which are synthesized from *Syzygium cumini*, exhibit concentration-, time-, and species-dependent antibiofilm activity against *Candida albicans, Staphylococcus aureus*, and *Staphylococcus epidermidis*, with effective concentrations ranging from 125 to 8,000 μg/mL (Bernardo et al., 2022). Similarly, *Gmelina arborea*-derived AgNPs and *Eugenia roxburghii*-derived AgNPs inhibited biofilm formation by disrupting bacterial colonization. Still, their efficacy was assessed using different assays, including Congo Red agar, colony counting, SEM, and cell-staining techniques, limiting direct quantitative comparison across studies (Chandrasekharan et al., 2022). Therefore, current evidence supports the antibiofilm potential of the plant-mediated nanoparticle; methodological heterogeneity and the limited evaluation of resistance-related endpoints prevent a direct conclusion that biofilm inhibition represents AMR mitigation (Hayat et al., 2022).

#### 4.4.4 Potential for selection of reduced nanoparticle susceptibility

Available evidence indicates that repeated exposure to sub-inhibitory concentrations of nanoparticles may select for bacterial adaptations associated with reduced nanoparticle susceptibility. The experiment shows that repeated exposure of *E. coli* to sub-inhibitory concentrations of AgNPs results in stable resistance, with a 30-fold increase in MIC, ranging from 5–150 mg/L. This resistance was linked to genetic changes affecting transport systems, flagellin production, and efflux mechanisms (Alhajjar et al., 2022). Similarly, the resistance associated with genetic adaptations accumulated during the prolonged AgNPs exposure in a long-term laboratory experiment (Graves et al., 2015). However, studies on chemically synthesized AgNPs and comparable long-term investigations on plant-mediated PNPs remain lacking (Graves et al., 2015; Alhajjar et al., 2022).

#### 4.4.5 Effects on horizontal gene transfer

Assessment of horizontal gene transfer (HGT) provides a more direct approach for investigating whether a nanoparticle intervention influences the dissemination of antimicrobial-resistance genes than measurement of antimicrobial activity alone. However, reduced bacterial growth or biofilm formation should not be interpreted as evidence of reduced HGT unless gene-transfer processes are experimentally measured (Lu et al., 2020). However, direct evidence of PNPs remains unavailable, so instead, experimental studies using AgNPs indicate the opposite effect. At environmentally relevant, non-lethal concentrations, AgNPs increased phage-mediated antibiotic resistance genes by up to 2.4-fold; by contrast, Ag+ increased them by 1.8-fold compared with the untreated control (Lu et al., 2020). Similarly, AgNPs increased phage-mediated antibiotic resistance gene transduction by 4.82-fold and extracellular transformation by 10.27-fold; in the same way, Ag^+^ increased transduction and transformation by 8.75-fold and 5.87-fold, respectively (Li et al., 2025). These effects were mainly caused by increased reactive oxygen species (ROS) production, damage to the bacterial membrane, and activation of the SOS stress response (Lu et al., 2020). Overall, the current evidence demonstrates that when AgNPs are present at sub-lethal concentrations, they can both promote and prevent horizontal gene transfer.

#### 4.4.6 Potential risks of metal-mediated co-selection of antibiotic resistance

A recent study demonstrated that low levels of AgNPs may unintentionally promote the spread of antibiotic resistance, although plant-mediated PNPs can kill harmful bacteria (Murray et al., 2024). A 5.7% variation in antibacterial resistance genes was explained by heavy metals alone, compared with 0.7% for antibiotics; meanwhile, mobile genetic elements and the composition of the bacterial community explained 12.4% and 21.9%, respectively (Mazhar et al., 2021). The effect of silver on antibiotic resistance is form-dependent, as repeated cyclic exposure to silver ions promotes adaptive antibiotic tolerance, whereas AgNPs did exhibit a detectable reduction in antibiotic resistance under the experimental conditions (Zhao et al., 2022). From the above evidence, it can be concluded that the influence of metal-based nanomaterials is highly dependent on environmental and exposure conditions, as well as the form of the metal.

## 5 Mechanisms, safety, and practical application of PNPs in the food system

### 5.1 Antimicrobial strategies

In traditional antimicrobial strategies, target-specific microbial pathways are targeted, allowing microorganisms to develop resistance over time (Blair et al., 2015). The antibacterial activity of β-lactam antibiotics interferes with peptidoglycan synthesis by interacting with penicillin-binding proteins (PBPs), thereby preventing crosslinking of the bacterial cell wall. Through enzymatic degradation, microbial resistance occurs, for example, when beta-lactamases hydrolyze the lactam ring (Bush and Bradford, 2016; Hense and Schuster, 2015). Chemical preservatives disrupt microbial metabolism by altering pH and enzyme activity, leading to a proton imbalance (El-Saber Batiha et al., 2021). In this regard, PNPs act via multiple biochemical pathways, controlled by redox reactions and oxidative stress mechanisms (Mondal et al., 2024). At the pathogen surface, the PNPs act as catalysts, promoting the formation of ROS via an electron-transfer reaction. The superoxide radical undergoes dismutation to form hydrogen peroxide (Mehta et al., 2022). This hydrogen peroxide takes part in a Fenton—type reaction to generate highly reactive hydroxyl radicals. These hydroxyl radicals induce lipid peroxidation in the microbial membrane. PNPs also bind with the thiol (-SH) groups of proteins, resulting in enzyme inactivation (Zhang et al., 2020). Moreover, nanoparticles can impair DNA function and induce oxidative damage, leading to strand breaks and replication (Godde et al., 2018).

Compared to conventional antimicrobials, which usually target a single biochemical pathway, PNPs affect multiple cellular processes (Mubeen et al., 2021). These include disrupting redox homeostasis, compromising membrane integrity, interfering with enzyme function, and damaging genetic material. Because PNPs simultaneously interact with multiple cellular pathways, they are less likely to promote resistance than single-target antimicrobial agents. However, direct experimental evidence demonstrating reduced resistance evolution during prolonged exposure remains limited, warranting further long-term investigation (Mondal et al., 2024).

### 5.2 Safety, toxicity, and regulatory considerations of PNPs

Although PNPs show potential antimicrobial activity, their safety and toxicity remain critical concerns for their application in the food system (Mondal et al., 2024). Higher nanoparticle concentrations may lead to oxidative stress in human cells, as in microbial cells (Liu and Qu, 2019). In addition, the generation of reactive oxygen species (ROS) can cause cellular damage in mammalian cells. A further challenge is the possible transfer of nanoparticles from packaging material into the food, potentially resulting in unintended human exposure (Ahmad et al., 2023). The interaction of PNPs with food components such as proteins, lipids, and carbohydrates may alter their phytochemical properties, thereby affecting both safety and efficacy (Mubeen et al., 2021). The use of nanomaterials in food systems is regulated by authorities such as the FDA and EFSA, which restrict their commercialization (El-Saber Batiha et al., 2021). In the successful implementation of nanotechnology-based food products, consumer perception and acceptance also play a significant role. Although preliminary studies have found biological responses to be favorable at certain doses and under experimental conditions, these results depend on the type of material, dose, duration, and experimental model used. Most of the available literature is based on short-term laboratory experiments, and long-term toxicity and bioaccumulation tests need to be conducted before food applications are recommended (Mubeen et al., 2021).

## 6 Application of PNPs in food safety

### 6.1 Antimicrobial food packaging system

The metal and metal oxide PNPs have attracted much interest in food safety applications due to their reported antimicrobial activity against certain selected Gram-positive and Gram-negative foodborne pathogens. However, antimicrobial effectiveness varies with nanoparticle properties and the type of microorganism tested (Ahmad et al., 2023). The use of nanoparticles in biodegradable polymer matrices ensures controlled diffusion and release kinetics of microbial agents, thus resulting in sustained Inhibition of foodborne pathogenic microorganisms (Bush and Bradford, 2016). Possible synergistic effects between plant phytochemicals and nanomaterials could increase antimicrobial potency and even allow lower nanoparticle concentrations in certain formulations, thereby achieving the desired antimicrobial effect. However, a decrease in toxicity should be demonstrated through toxicology evaluation specific to a particular formulation (Mubeen et al., 2021). In a nutshell, despite promising antimicrobial performance, most packaging studies have been conducted under controlled laboratory conditions or food simulants. However, the validation under commercial processing, storage, and distribution conditions remains limited (El-Saber Batiha et al., 2021; Pandian et al., 2023; Demirbas and Karsli, 2025; Trak et al., 2025).

### 6.2 Surface sanitation and biofilm control in the food processing environment

PNPs based coating approach for surface disinfection. The spray coating approach has the potential to change the way that we clean the food processing surfaces, especially for controlling multidrug-resistant contamination on the surface, where biofilms form extensively. The PNPs would be capable of diffusing through the EPS matrices, degrading their architecture via oxidative breakdown processes, and arresting auxin-sensing signaling pathways. With a combined effect against multidrug-resistant pathogenic and non-pathogenic bacterial strains resistant to conventional disinfection technologies, through a multitarget mechanism of Inhibition of various biochemical and biological pathways. Nanocoating may be applied to any surfaces and any apparatus used throughout the food handling systems, such as working platforms, machines, storage rooms and all the equipment on which the raw materials or the end product might be transported to minimize surface contamination and maximize hygienic surfaces (Mu et al., 2023).

### 6.3 Application of PNPs in food preservation

Natural antimicrobials have emerged as a sustainable alternative to synthetic preservatives, and they are gaining attention in modern food preservation systems (Pisoschi et al., 2018). These compounds, such as plant extracts, essential oils, organic acids, and bacteriocins, demonstrate broad-spectrum antibacterial activity against Gram-positive and Gram-negative foodborne pathogens (Gyawali and Ibrahim, 2014). They disrupt the microbial cell membrane, alter intercellular pH, inhibit enzymes, and impair nucleic acid synthesis (Pisoschi et al., 2018). Currently, research focuses on improving the stability and efficiency of natural antimicrobials through encapsulation techniques, including nanoencapsulation and emulsion-based delivery system (Xu et al., 2022). Their long-term antimicrobial effectiveness is enhanced when these compounds are incorporated into active food packaging materials (Mehta et al., 2022). Furthermore, the integration of antimicrobials with nanotechnology, especially PNPs, has demonstrated synergistic effects that enhance antimicrobial performance and overcome limitations such as low bioavailability (Mubeen et al., 2021). These approaches align with the growing consumer demand for clean-label, minimally processed, and chemical-free food production, supporting their adoption in a sustainable food system (Nile et al., 2020; Barciela et al., 2023; Kaushal et al., 2024). The literature on the main studies presented in **Table 6** demonstrates the promising potential of PNPs for use in food preservation, active food packaging, and microbial Inhibition under practical food storage conditions. It is essential to carefully consider these results into account, since the antimicrobial properties of nano systems as determined by agar diffusion or broth microdilution methods do not necessarily translate to their behavior in the food matrix. Indeed, food constituents such as proteins, lipids, carbohydrates, salts, organic matter, pH, and water activity may affect dispersion and aggregation, metal ion leaching, and interactions between nanoparticles and microbes. Therefore, the antimicrobial tests conducted in laboratory conditions can serve only as preliminary data, while those performed within a food system under practical conditions are more reliable (Nayab and Akhtar, 2023; Pandian et al., 2023; Likhar et al., 2026).

**Table 6:** Representative primary studies evaluating PNPs in real food systems for antimicrobial activity and food preservation.

Food matrix	PNP composition & plant source	Application strategy	Target microorganisms	Experimental design & NP loading	Antimicrobial/shelf-life outcomes	Safety/limitations	Reference
Strawberry	AgNP–cellulose sponge; plant source: NR	Active antimicrobial packaging	*E. coli*, *S. aureus*, *R. stolonifer*	Simulated transportation storage; loading: NR	Antibacterial/antifungal activity; shelf life extended to 12 days with reduced microbial invasion	Mechanical protection assessed; sensory NR; single-fruit model; scale-up not validated	(Zhang et al., 2022)
Strawberry	Plant-mediated AgNP–PLA film; mango peel extract	Active packaging film	*E. coli*, *S. aureus*	Packaging storage study; loading: NR	>95% bacterial inhibition; deterioration prevented for 7 days; barrier properties improved	Cell viability >80%; sensory NR; single food matrix	(Cheng et al., 2021)
Strawberry	AgNP–starch coating; plant source: NR	Edible coating	Natural spoilage microflora	Refrigerated storage; loading: NR	Reduced microbial spoilage; improved storage time and fruit quality	Quality attributes assessed; migration not extensively evaluated	(Taha et al., 2022)
Shrimp	Nanoparticle–alginate coating; grapefruit seed extract	Edible coating	Natural spoilage microorganisms	Cold storage at 4 °C; loading: NR	Delayed microbial growth; shelf life extended to 8 days; improved TVB-N, pH and sensory quality	Sensory evaluation performed; not a metallic phyto-nanoparticle	(Baek et al., 2021)
Broiler breast meat	Pullulan film with curcumin-mediated AgNPs, pullulan-mediated AgNPs and mixed AgNPs; *C. longa*	Bi-layer edible film; vacuum packed	Not specified	4 ± 1 °C, 0–14 days; 2% AgNPs	Maintained physicochemical quality and improved oxidative stability	No microbial challenge, migration, sensory or toxicological assessment; scale-up not validated	(Khan et al., 2022)
Fior di latte cheese	Ag–montmorillonite (Ag-MMT) in sodium alginate; plant source: not applicable	Antimicrobial coating + MAP	*Pseudomonas* spp., coliforms, LAB, yeasts, molds, total microbes	5, 10 and 15 mg/mL Ag-MMT; MAP; 10 °C, 7 days	Reduced microbial growth; improved storage stability	Not plant-mediated; therefore outside the strict PNP scope	(Gammariello et al., 2011)

NR, Not Reported.

Although several studies demonstrate promising antimicrobial and shelf-life benefits in real food matrices, the studies are not directly comparable because of differences in food composition, PNP formulation, dosage, storage conditions, microbial populations, and outcome measurements. Consequently, the observed benefits should be considered system-specific rather than evidence of consistent efficacy across food categories. Furthermore, limited information on nanoparticle migration, exposure, toxicity, and industrial-scale performance in several studies restricts broader conclusions regarding practical applicability.

## 7 Challenges and safety concerns of PNPs

### 7.1 Regulatory consideration

The main obstacle to the regulatory approval of PNPs is the absence of specific guidelines and standard assessment procedures. In the absence of standard procedures, PNPs are assessed depending upon the criteria established for the assessment of conventional foods, food additives, and food contact materials. For food contact materials, special consideration is given to the particle size and shape, surface chemistry, potential release of metal ions, and stability of the materials in food. The migration of PNPs from active food packaging and release of PNPs from edible coatings should be evaluated using worst-case scenarios, and exposure of the end-users should be evaluated. In the absence of uniform regulations, it is extremely important to evaluate the exposure of the end users to PNPs and perform comprehensive assessment of the PNPs to establish their safety for long-term use.

International trade is more difficult than ever due to the varied regulation across regions. It is necessary to establish and maintain clearly-organized regulations that enable approvals and guarantee safe use (Hamburg, 2012; Wang et al., 2021).Evaluating the safety of PNPs for use in the food industry is complicated for a number of reasons. These include the composition and physical properties of the nanoparticles, the proposed use of the nanoparticles, the way in which the food containing the nanoparticles will be prepared and consumed, and the jurisdiction in which the assessment is performed. The safety of a particular type of nanoparticle for a particular use in a food may also depend on the nature of the food, the methods used to process and preserve the food, and other factors (Hamburg, 2012). The European Food Safety Authority (EFSA) recommends that nanosize components of materials used in the food and animal feed industry be assessed for safety. This includes not only the nanomaterials, but also other components of the feed or food that may consist of small particles (Gupta et al., 2024; Ahmed et al., 2026).

### 7.2 Environmental impact

The nanoparticle, when released into the environment, may directly or indirectly affect soil microorganisms and the ecosystem. Besides that, they can accumulate in soil and water systems, disrupting the microbial ecosystem. Moreover, nanoparticles may also be taken up by plants and enter the food system (Gao et al., 2023). It also has the potential to reach critical levels in water, algae, fish, and other living inhabitants, consequently to minimize risks, there will be a need for a disposal system and an environmental management system. Although, in general, plant mediated methods were considered environmentally preferable compared with to traditional chemical methods for nanoparticle preparation, classifying ‘PNPs’ as ‘environmentally friendly’ is not fair (Barciela et al., 2023).

Many aspects should be considered for plant - mediated methods: land consumption, water resource use by the plant, fertilizer input, pesticide input, production yield of biomass, type of organic solvent, reaction temperature, energy used in shaking or heating and volume of water and chemicals employed in the washing step process, including purification and centrifugation process. In the accidental released it of metals (AgNPs, ZnO NPs and CU O NPs) into water or soil, these particles would adversely affect microbes and fish through biological processes, thereby impacting the health of the entire ecosystem. One important advantage of plant-derived nanoparticles is their ability to replace chemical reducing agents. However, overall sustainability also depends on the environmental fate of nanoparticles, energy consumption, water purification, extraction, and biomass production (Atofarati et al., 2026). So, a lifecycle assessment, an environmental risk assessment, and sustainable waste management approaches should be conducted before determining whether plant-mediated PNPs are environmentally friendly for large scale food applications (Gao et al., 2023). Subsequent work should combine a life-cycle assessment (LCA), a green chemistry indicator and an ecotoxicological assessment to compare plant-mediated nanoparticles to conventional nanotechnologies objectively (Barciela et al., 2023; Shakeel et al., 2025).

## 8 Research gaps, limitations and future prospects

Although PNPs have demonstrated promising antimicrobial properties, several limitations currently restrict their translation into practical food-safety and preservation applications. These limitations are associated with limited validation in real food systems, methodological variability, incomplete mechanistic understanding, insufficient safety and exposure data, and challenges related to scale-up and regulatory evaluation.

### 8.1 Limited validation in real food systems

Most PNPs studies involve lab conditions and fewer studies assess PNP performance in food matrices. Studies have not systematically assessed the impact of PNPs on dairy products, meat, seafood, fruits, vegetables, and processed food (Duda-Chodak et al., 2023). The impact of PNPs in food is still underexplored and is highly dependent on food composition and the conditions of food processing.

Food composition and food processing conditions impact the behavior of nanoparticles, including their stability and interactions with food components, and can also affect nanoparticles’ antimicrobial activity (Wang et al., 2017). It is therefore important to validate the performance of PNPs in food, especially if an extension in food shelf life is reported (Jangid et al., 2025).

### 8.2 Standardization and reproducibility of PNPs

A high degree of variability exists for reported PNP formulations due to differences in plant sources, extraction methods, the kind of phytochemicals, physicochemical properties of nanoparticles, and conditions of synthesis. Other variability is due to differences in the microbial strains, and the conditions of the laboratory. The basis for the antimicrobial activity and the mechanism of action are highly variable among studies (Jangid et al., 2025). Variability among studies limits the comparability and reproducibility of the research. Uniform methods are needed to facilitate comparison among studies (Demirbas and Karsli, 2025).

### 8.3 Unresolved antimicrobial mechanisms and AMR relevance

Despite many investigations, the contributions of intact nanoparticles, released metal ions, reactive oxygen species, and surface-bound phytochemicals to antimicrobial activity are not completely clear. The current manuscript describes that antimicrobial effects may come from (i) interactions between nanoparticles and microbial cells, (ii) metal ions released from nanoparticles, and (iii) surface-associated phytochemicals. Therefore, the comparison of results with metal ions and phytochemical controls is essential for a plausible explanation.

This also applies to antimicrobial resistance (AMR). PNPs exhibit antimicrobial activities against a number of foodborne and resistant microorganisms, but evidence to support their application reduces the development and spread of resistance and resistance determinants in food systems is still limited (Barciela et al., 2023; Kaushal et al., 2024). Therefore, antimicrobial activity against resistant microorganisms should not be interpreted to mean PNPs mitigate AMR.

### 8.4 Safety, migration and environmental considerations

Safety assessment continues to be a major obstacle for the use of PNPs in food systems. Evidence of safety is usually from laboratory studies of short-term duration. Information about long term toxicity, bioaccumulation, dietary exposure, and migration of nanoparticles is scanty. The literature also points to the need for food-based toxicological assessment and migration from food contact materials (Demirbas and Karsli, 2025; Jangid et al., 2025).

The safety of PNPs can be influenced by the interactions of PNPs with food components, and consequently, the properties and safety of the PNPs may be altered. Thus, the safety of PNPs should be evaluated based on the final formulation and the conditions of use, and not under simplified conditions, which are often used in the laboratory. An environmental assessment is essential because the use of plant extracts does not, in itself, establish that the resulting PNPs are environmentally safe. The assessment should consider the life cycle of the PNPs as well as their ultimate fate (Mohanta et al., 2017; Kaushal et al., 2024). Life-cycle assessment, environmental-risk assessment, and ecotoxicological studies should be part of a comprehensive assessment of the overall sustainability of plant-mediated PNPs (Barciela et al., 2023; Shakeel et al., 2025).

### 8.5 Scale-up, regulatory requirements and commercialization

There are additional challenges when moving from lab-scale synthesis to industry. Variability of plant extracts can result in variability in the synthesis of NPs. Lack of standardization of methods can impact reproducibility and scalability (Borehalli Mayegowda et al., 2023). The large-scale production of NPs necessitates use of stable and consistent starting raw materials; control over the synthesis parameters and conditions; efficient purification and separation; reproducible and consistent NPs; and management of energy, water and waste. In addition, regulatory approval of food contact, migration, long term toxicity and processing evaluations also impact commercialization. Consumer acceptance, confidence and trust may also influence incorporation of nanotechnology in food applications. While some nanotech-based products in the market have shown positive outcomes in their application, the same cannot be said for plant mediated PNPs (Mondal et al., 2024). Important barriers still remain as regulatory and large-scale challenges.

### 8.6 Future research priorities

Future studies should focus on designing a more comprehensive and integrated framework that incorporates food validation and food safety, in addition to design, mechanism, translation and the evaluation of synthesis and safety. Special attention should be given to synthesis standardization and characterization, performing mechanistic studies with sufficient controls, validation in real food matrices, migration and exposure assessment, toxicological studies and evaluation of the impacts on the environment, and assessment under actual processing and storage conditions.

Other advanced methodologies such as artificial intelligence (AI) and machine learning may provide possible synthesis of nanoparticles and predict relationships between the parameters of synthesis and the behavior of nanoparticles and antimicrobial efficiency (Dheyab et al., 2025). These methods could reduce the time and effort for developing formulations in laboratory experiments.

## 9 AI-assisted design and prediction of PNPs

The use of Artificial intelligence (AI) and Machine learning (ML) is proving to be an effective method for shifting the development of antimicrobial nanoparticles from an empirical to a data-driven framework. Within the context of PNPs, information associated with the species of plants, chemistry of phytochemicals, extraction, metal precursors, and nanoparticle synthesis along with the characteristics of microbes and the antimicrobial activities, can be processed by AI-driven systems. Such systems may help describe and predict the optimum design of nanoparticles. Given the multitude of factors associated with PNPs, developing an optimum nanoparticle formulation would likely involve numerous “trial-and-error” experiments. AI-based systems may help condense the number of experiments required to develop optimized nanoparticle formulations. Currently, this approach has already been demonstrated to be feasible using experimental evidence. Saadat et al. (2022) trained some ML models for predicting the antibacterial activities of green-synthesized AgNPs. Their dataset was built from 100 published papers and added some experimental variables (such as the volume and amount of plant extract, solvent volume, concentration and the volume of AgNO_3_ and reaction time and temperature, the concentration of the precursor, the concentration of bacteria, and the concentration of the synthesized nanoparticles). The models were tested against *E. coli*, *P. aeruginosa*, *S. aureus*, and *K. pneumoniae*, and the size and concentration of nanoparticles were identified as the most important predictors. In the tested models, the highest performance was obtained by XGBoost for predicting *P. aeruginosa* (R² = 0.84), and the lowest reported R² was 0.52 for *S. aureus*, which was obtained by AdaBoost.

The nano informatics studied by Mirzaei et al. (2021), which included a larger dataset, further validated the use of ML for predicting antibacterial activities of nanoparticles. Their dataset consisted of 1,176 observations obtained from 60 published papers, with a final dataset of 436 observations including the predictors of nanoparticle type, shape and size, coating, dose and exposure time, and bacterial species and Zone of inhibition (ZOI). The Random Forest model yielded R² = 0.78. Among the features in the model, the most important was the size of the nanoparticle core, followed by the exposure dose and bacterial species.

Finally, yet another study performed in 2026 claimed a more advanced method for predicting the antimicrobial activity of AgNPs, based on a liquid-state machine, together with particle shape, core size and UV-Vis characterization, and the ZOI obtained from experiments. The fully integrated model showed an R² of 0.956, an RMSE of 1.151, and an MAE of 0.486. SHAP analysis placed the concentration and duration of exposure as the main contributors toward antimicrobial activity, whereas the microbial species, the capping agent, and the reducing agent were of lower importance. These findings show the extent to which integrated information on physicochemical nanoparticle data and experimental antimicrobial data can contribute to the development of interpretable predictive models (Dechakupt et al., 2026).

The need for such an approach is apparent when considering some of the complexities of experimental antimicrobial data. As an example, Loo et al. (2018) reported their study of using green-synthesized AgNPs as AgNPs extracted from Pu-erh tea leaves against foodborne *E. coli*, *K. pneumoniae*, *S. Typhimurium*, and *S. Enteritidis*. The nanoparticles exhibited an average size of 4.06 nm, and the MIC values were in the range of 3.9-7.8 μg/mL against the different organisms examined. Time-kill tests showed a reduction of 3 logs (99.9%) of the cells in 1–2 hours. The types of data reported herein, which include the plant source, nanoparticle characteristics, different bacteria, MIC/MBC, and time-kill data, are the types of validated data that should be incorporated into AI models for predicting foodborne pathogen control. An AI/ML-assisted framework for the rational design and optimization of PNPs is illustrated in **Figure 6**.

**Figure 6:**
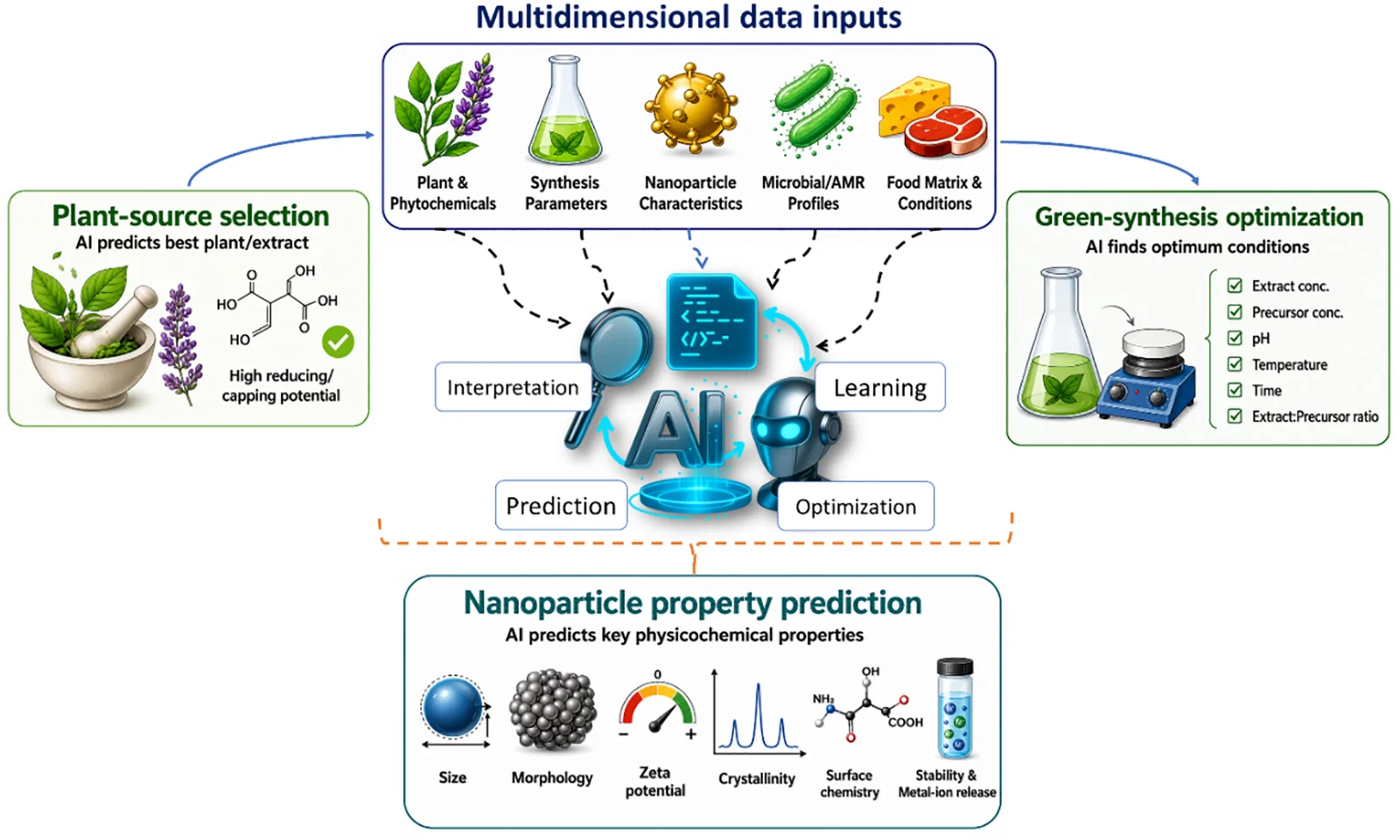
AI/ML-assisted framework for the rational design and optimization of PNPs.

## 10 Conclusion

Studies show that phyto-nanoparticles (PNPs) can be effective against foodborne pathogens and can be used in active food packages, edible coatings, and other food safety measures. The studies reviewed in this paper have shown that synthesizing nanoparticles using plants can produce antimicrobial nanoparticles. There are, however, numerous factors that can influence the efficacy of these nanoparticles including the plant source and phytochemicals, the microorganism used in the study, the assay methods and the conditions under which the study was conducted. Therefore, studies cannot always compare and generalize their findings. The studies also suggest that the use of PNPs does not impact antimicrobial resistance. Some studies have shown that phytochemicals can impact foodborne pathogens; however, the focus of these studies has been on the mechanisms and pathways of antimicrobial resistance rather than the behaviors of PNPs. Finally, the evidence is lacking regarding the role of other reactive species and ions, especially metal ions, in the context of food systems. While it is clear that PNPs can kill antibiotic-resistant microorganisms, this does not mean that using PNPs to kill microorganisms will help solve the problem of antibiotic resistance. More studies will be needed to understand the role of PNPs and other reactive species in killing microorganisms. There are a few studies that point to the potential use of PNPs to kill or remove microorganisms from food and to slow the spoilage of food. However, the results from the lab are usually hard to implement in the food industry. Hence, there is a need for more studies to evaluate the safety and health effects of long-term use, the environmental impact of large-scale production, and the potential use of PNPs in the food industry.

## Glossary

Ag-MMT: Silver–montmorillonite
AgNPs: Silver Nanoparticles
AI: Artificial Intelligence
AMP: Antimicrobial Peptides
AMR: Antimicrobial Resistance
AuNPs: Gold Nanoparticles
CDC: Centers for Diseases Control
CFU: Colony forming units
CRISPR: Clustered Regularly Interspaced Short Palindromic Repeats
CuO: NPs Copper Oxide Nanoparticles
DNA: Deoxyribonucleic Acid
EFSA: European Food Safety Authority
EPS: Extracellular Polymeric Substance
FDA: Food and Drug Administration
FTIR: Fourier Transform Infrared Spectroscopy
HGT: Horizontal Gene Transfer
HPLC: High performance liquid chromatography
HUS: Hemolytic uremic syndrome
ICP–MS: Inductively coupled plasma–mass spectrometry
LAB: Lactic acid bacteria
LC-MS: Liquid chromatography–mass spectrometry
MAP: Modified atmosphere packaging
MBC: Minimum bactericidal concentration
MDR: Multidrug Resistance
MIC: Minimum Inhibitory Concentration
ML: Machine Learning.
MRSA: Methicillin-resistant Staphylococcus aureus
NP: Nanoparticle
PBPs: Penicillin-Binding Proteins
PDI: Polydispersity index
PNPs: Phytonanoparticles
PRISMA: Preferred Reporting Items for Systematic Reviews and Meta-Analyses
ROS: Reactive Oxygen Species
SEM: Scanning Electron Microscopy
TEM: Transmission Electron Microscopy
TVB-N: Total volatile basic nitrogen
TVC: Total viable count
UV–Vis: Ultraviolet–Visible Spectroscopy
XRD: X-ray Diffraction
ZnO: NPs Zinc Oxide Nanoparticles
ZOI: Zone of inhibition
